# Imaging of fluorescence anisotropy during photoswitching provides a simple readout for protein self-association

**DOI:** 10.1038/s41467-019-13843-6

**Published:** 2020-01-07

**Authors:** Namrata Ojha, Kristin H. Rainey, George H. Patterson

**Affiliations:** Section on Biophotonics, National Institute of Biomedical Imaging and Bioengineering, National Institutes of Health, Bethesda, MD 20892 USA

**Keywords:** Wide-field fluorescence microscopy, Biological fluorescence

## Abstract

Monitoring of protein oligomerization has benefited greatly from Förster Resonance Energy Transfer (FRET) measurements. Although donors and acceptors are typically fluorescent molecules with different spectra, homo-FRET can occur between fluorescent molecules of the same type if the emission spectrum overlaps with the absorption spectrum. Here, we describe homo-FRET measurements by monitoring anisotropy changes in photoswitchable fluorescent proteins while photoswitching to the off state. These offer the capability to estimate anisotropy in the same specimen during homo-FRET as well as non-FRET conditions. We demonstrate photoswitching anisotropy FRET (psAFRET) with a number of test chimeras and example oligomeric complexes inside living cells. We also present an equation derived from FRET and anisotropy equations which converts anisotropy changes into a factor we call delta r FRET (drFRET). This is analogous to an energy transfer efficiency and allows experiments performed on a given homo-FRET pair to be more easily compared across different optical configurations.

## Abstract (web summary)

Performing homo-FRET measurements in cells using a fluorescence microscope is challenging, especially when using high numerical aperture objective lenses. Here the authors present a method for improved homo-FRET measurements based on anisotropy changes in photoswitchable fluorescent proteins.

## Introduction

The interaction and oligomerization of proteins are often important factors in activation or amplification of cellular signaling pathways. As a consequence, biologists continue to investigate protein–protein interactions to better understand the pathways, to discover new interacting partners, and develop small molecules which influence the behavior of proteins of interest. Numerous imaging methods have been developed to monitor protein–protein interactions on the single cell level. Among these are Förster Resonance Energy Transfer (FRET) methods which rely on excitation of a donor molecule which transfers excited state energy to an acceptor molecule^1^.

FRET experiments are typically performed using different color donor and acceptor molecules with the donor emission spectrum overlapping with the absorption spectrum of the acceptor and these are often referred to as hetero-FRET experiments^2^. However, the emission spectra of many fluorescent molecules display overlap with their own absorption spectra. Thus, if the two molecules are close enough, the exited state energy from one molecule can be transferred to the other via homo-FRET. The accepting molecule in this example has an emission spectrum, quantum yield, and fluorescence lifetime indistinguishable from the donating molecule, so homo-FRET cannot be detected as in a typical FRET experiment^1,2^. On the other hand, the orientation of the dipoles of the donating molecules and accepting molecules can differ and the fluorescence emission from accepting molecules will be polarized differently than the emission from donating molecules. These changes can be observed by anisotropy measurements which require excitation with polarized light and monitoring of the parallel and perpendicular polarized emission intensities.

Time-resolved measurements of anisotropy provide the most information about the molecule rotational dynamics and energy transfer between fluorophores^2,3^. These experiments provide insight into the molecular motion of tagged fluorophores, readouts of homo-FRET^4^, and insight into the oligomeric state of molecular complexes or clusters^5–10^ in some cases based on the theoretical underpinnings by Runnels and Scarlata^11^. The required instrumentation and imaging skills for such an advanced technique may not be available to most biologists, but a concise book chapter on the subject written for novices is recommended for those wishing to learn more^12^. On the other hand, requirements for steady-state anisotropy imaging are not particularly onerous. The applications of steady-state anisotropy imaging are quite vast, especially when considering the use of conventional fluorophores^11,13,14^. The introduction of GFP^15^ further expanded these applications, which have often centered on studies of protein oligomerization^16–18^ or protein conformation changes during receptor or sensor activation^19,20^. However, making these measurements inside cells using a fluorescence microscope can still present challenges. For instance, the steep collection angles afforded by high numerical aperture (NA) objective lenses result in mixing of polarizations and leads to decreased anisotropy as well as decreased changes in anisotropy due to homo-FRET^21,22^. As a consequence, anisotropy measurements are often made using low NA objective lenses to limit polarization mixing at the expense of optical resolution^22^.

Since comparison of anisotropy values from control experiments where the homo-FRET does not occur are often required to reveal the presence of homo-FRET and thus protein oligomerization^1,2^, steady-state measurements may also be limited when studying obligate oligomers. For instance, when a protein is labeled or expressed in a cell, the measured anisotropy will be a combination of steady-state anisotropy of the protein of interest and any homo-FRET that may be occurring. Thus, the anisotropy in the absence of homo-FRET may not be easily determined for proteins always found in oligomeric complexes. Moreover, the anisotropy of a monomeric tagged protein may not be the same as the anisotropy of an oligomeric tagged protein even in the absence of homo-FRET.

These limitations as well as our own experience with imaging homo-FRET using steady-state anisotropy led us to explore new approaches. Recently we developed photoswitching Förster Resonance Energy Transfer (psFRET), a technique for imaging interactions between two different fluorescent proteins^23^. Here, we introduce a related method which we refer to as photoswitching Anisotropy FRET (psAFRET), that is similar to monitoring the anisotropy of fluorescent samples as they are photobleached^4,6,16^. By extending this approach to the use of photoswitchable fluorescent proteins^24^ and monitoring anisotropy as they photoswitch off, we can avoid high levels of irradiation associated with photobleaching, we can photoswitch molecules off much faster than photobleaching, and we can photoswitch the proteins back on to repeat the experiment. Here, we present studies developing and testing psAFRET as a reliable option for monitoring homo-FRET and demonstrate its use to monitor oligomerizing proteins inside living cells. Moreover, we present a derivation to convert anisotropy changes into values analogous to hetero-FRET efficiencies. Similar to well-defined hetero-FRET determinations^25^, a quantitative value reporting an efficiency is advantageous for homo-FRET studies since it should be reproducible across instruments and laboratories when imaging the same molecules.

## Results

### Photoswitching and photobleaching AFRET principle

Anisotropy FRET (AFRET) microscopy relies on preferential excitation of molecules with dipoles oriented parallel with the polarization of the excitation light. The sample anisotropy is determined by collection of fluorescence emission through polarizing filters oriented parallel (I_||_) and perpendicular (${\mathrm{I}}_ \bot$) to the excitation polarization followed by calculation using following Eq. (1).

1 $$r = \frac{{I_{||} - I_ \bot }}{{I_{||} + 2I_ \bot }}$$

For small fluorescent molecules, their rotational correlation times are normally much faster than their fluorescence lifetime, so the dipole of the molecule can adopt a large range of random orientations before fluorescence is emitted^1,2^. Thus, the polarization of the fluorescence emission will differ markedly from the polarization of the excitation light and give a low anisotropy. On the other hand, fluorescent proteins typically have rotational correlation times much slower than their fluorescence lifetimes which leads to less depolarization and thus fluorescence emission polarization closer to that of the excitation light^1,2^. Importantly for our application, fluorescent proteins have a relatively a high intrinsic anisotropy value and homo-FRET can be detected by decreased anisotropy.

**Fig. 1:**
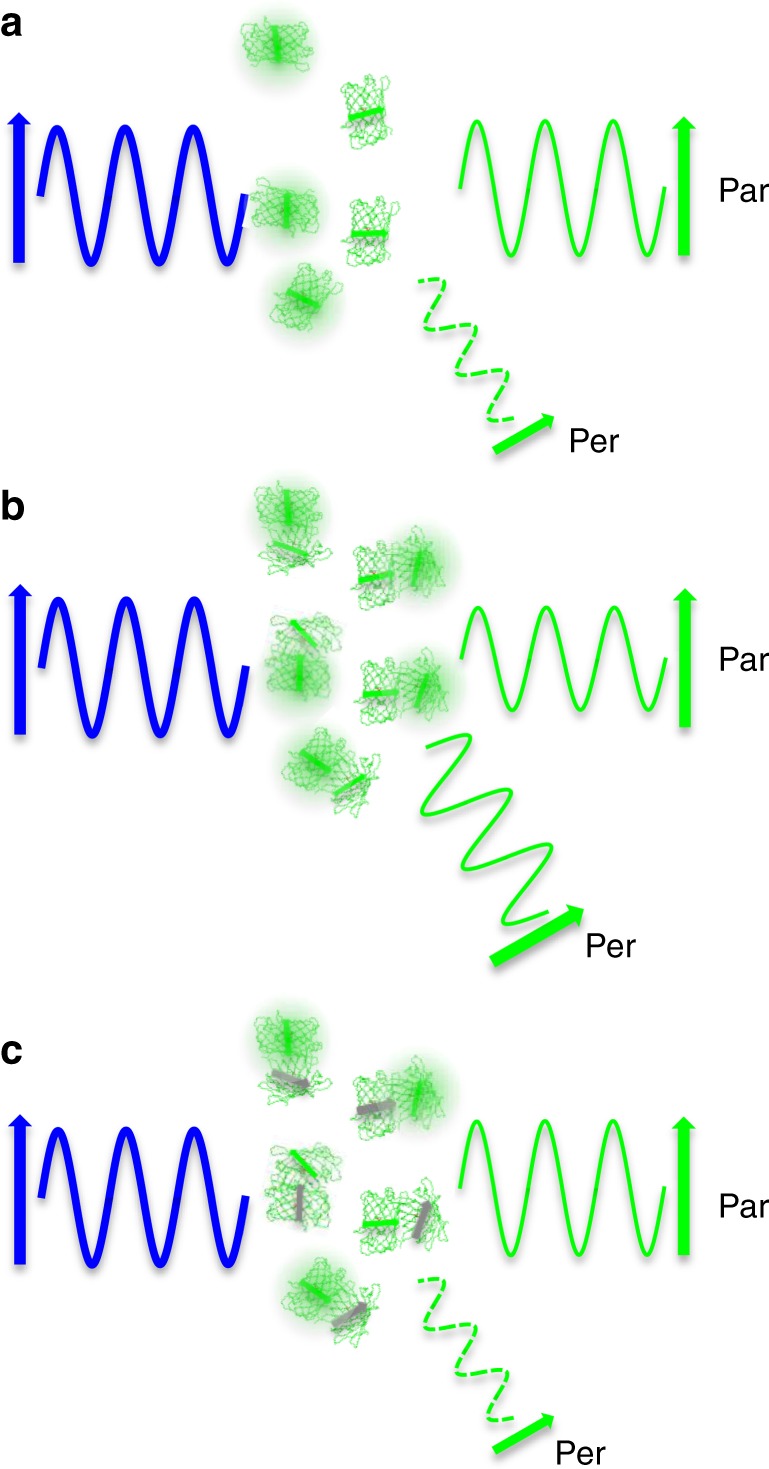
Principle of photoswitching anisotropy FRET. **a** Illumination of a population of sparse, non-interacting fluorescent protein molecules with polarized light (shown in blue) will lead to preferential excitation of molecules with dipoles oriented with the excitation polarization. The subsequent fluorescence emission (shown in green) detected in channels selectively filtered for parallel (par) or perpendicular (per) polarized light will produce a characteristic parallel to perpendicular ratio. **b** If the molecules are bound together close enough to homo-FRET, the molecules preferentially excited with polarized blue light can transfer their excited state energy to molecules which have dipoles randomly oriented with respect to the excitation polarization. This leads to an increase in perpendicular polarized emission at the expense of parallel polarized emission. **c** Fluorescent molecules in a complex which are photoswitched off or photobleached become unable to accept energy from an excited partner and the parallel to perpendicular ratio increases.

Given the similar principles of photobleaching AFRET and photoswitching AFRET to detect changes in anisotropy, we discuss them together. Consider a population of fluorescent proteins localized sparsely enough to make homo-FRET improbable (Fig. 1a). Under intense polarized illumination, these molecules will photoswitch or photobleach to an off or dark state which no longer absorbs nor emits light. Although the total fluorescence of the population decays over time, the decrease in the parallel and perpendicular channels will be proportional and the anisotropy will not change. Next consider a population of dimerized fluorescent proteins under polarized illumination (Fig. 1b). Since the molecules are no longer localized sparsely, the excited state energy of one molecule in a dimer can transfer to the other molecule in that dimer. In this case, the signal will decrease in the parallel channel, increase in the perpendicular channel, and the anisotropy will be lower when all of the molecules are on because the accepting molecule dipoles are likely oriented differently than their donating companions. However, as molecules within the dimers turn off, they can no longer absorb light, emit fluorescence, transfer excited state energy, nor act as energy transfer acceptors (Fig. 1c). The overall fluorescence will decrease, but the signals in the two detection channels will not maintain the same proportionality over the photoswitching cycle. The relative proportion of the perpendicular channel signal will decrease, the relative proportion of the parallel channel signal will increase, and the calculated anisotropy will increase because fewer of the molecules in the on state will also have a partner in the on state which can accept the energy. In summary, while all interacting molecules are fluorescent, they can energy transfer between each other and produce a decreased anisotropy. As the population of molecules are photoswitched off or photobleached over time, the measured anisotropy will increase to the point where it reports on conditions with little or no FRET. Therefore, the anisotropy values under homo-FRET and non-FRET conditions can be estimated within the same sample using psAFRET.

Although homo-FRET studies typically report changes in anisotropy, reporting efficiencies would be beneficial since these do not rely on specific instrument parameters and could be more easily reproduced across laboratories. To this end, several previous publications have discussed quantitative relationships between steady-state anisotropy measurements and energy transfer efficiency^26–29^. In some of his seminal studies, Weber described the relationship between polarization, fluorophore concentration, and the average fluorophore distance^26^. Since we seldom have an accurate estimate of the fluorophore concentration in our imaging experiments, we do not attempt to utilize Weber’s equation. With several equations available, we are unclear which should apply to our experiments. In Supplementary Fig. 1, we graphed the energy transfer (*E*) as a function of *r*_et1_, which is the anisotropy during energy transfer and *r*_et0_, which is the anisotropy in the absence of energy transfer. Unfortunately, these equations produce disparate values over the anisotropy range possible for an isotropic population of molecules (−0.2–0.4). More importantly, those equations^26–29^ are derived with the assumption that the average value of *κ*^2^, the dipole orientation factor, is equal to 2/3. While this is a reasonable assumption for small fluorophores with rotational correlation times much faster than their fluorescence lifetime, this is not valid for much larger fluorescent proteins which rotate very little during their fluorescence lifetimes^30,31^. Thus, it is unclear if these equations can be properly applied to data from our psAFRET method.

Therefore, we developed a separate approach to convert anisotropy measurements into a term akin to an energy transfer efficiency. For this, we consider homo-FRET as a sensitized emission experiment which normally utilizes two different types of fluorophores as the donor–acceptor pair and relies on measuring increased fluorescence in the acceptor channel. Here, we assume the parallel and perpendicular emission channels to be analogous to the donor and FRET emission channels, respectively, of a normal hetero-FRET imaging experiment. The discussion and derivation are available in the supplementary information (see Supplementary Note 1) and the resulting Eq. (2) can be used to convert changes in anisotropy to drFRET efficiencies.

2 $${\mathrm{drFRET}} = \frac{{6\left( {r_{{\mathrm{et}}0} - r_{{\mathrm{et}}1}} \right)}}{{1 + 8r_{{\mathrm{et}}0} - 4r_{{\mathrm{et}}1} + 4r_{{\mathrm{et}}0}r_{{\mathrm{et}}1}}}$$

The anisotropy for the control (*r*_et0_) should represent the anisotropy of the molecule of interest in the absence of homo-FRET. This seems obvious, but it may not be practical to determine this value for some molecules. For instance, a fluorescent tagged protein may have different anisotropies as a monomer versus a dimer even in the absence of FRET between the fluorescent molecules since the rotational dynamics of the dimer might be different compared with the monomer. In this example, the dimer in the absence of FRET (*r*_et0_) would have a higher anisotropy than a monomer. Therefore, while the presence of homo-FRET in a dimer would reduce the anisotropy to *r*_et1_, comparing this to the *r*_et0_ value determined from a monomer would lead to an underestimation of the true energy transfer.

On the other hand, for psAFRET experiments, the control value (*r*_et0_) is estimated for each sample regardless of the oligomerization characteristics of the protein of interest. The drFRET values for the full range of possible *r*_et0_ and *r*_et1_ values in an isotropic population of molecules shows the dependence not only on the change in anisotropy (delta r) but also on the measured anisotropy values under FRET and non-FRET conditions (Supplementary Figs. 1 and  2).

### Photoswitching AFRET imaging of Dronpa tandem dimers

**Fig. 2:**
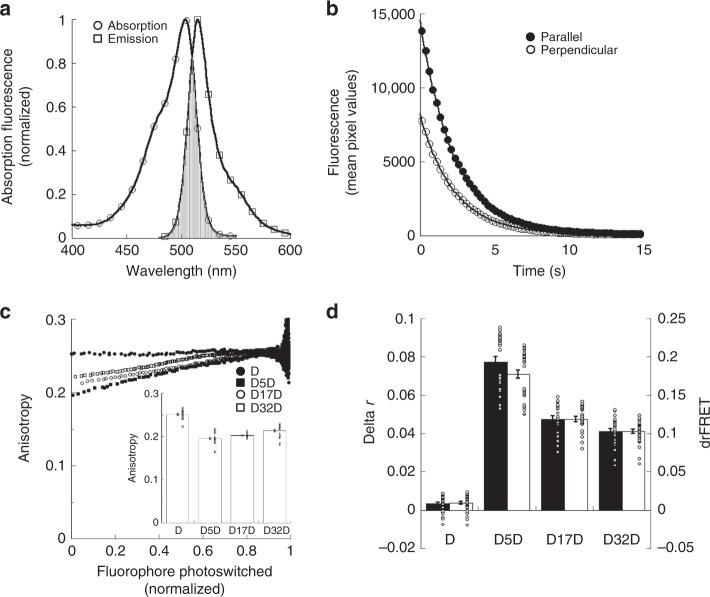
psAFRET measurements of Dronpa fluorescent protein dimers. **a** Absorption (open circles) and emission (open squares) spectra of purified Dronpa protein are shown in the on state. The spectral overlap between the absorption and emission are shown by the gray area. **b** A cell expressing Dronpa was excited using 488 nm excitation and imaged with parallel and perpendicular emission channels. The fluorescence intensities in the parallel (black circles) and perpendicular (white circles) channels are displayed as Dronpa is photoswitched off. **c** COS-7 cells expressing Dronpa (black circles), D5D (black squares), D17D (white circles), or D32D (white squares) were imaged and photoswitched. The anisotropy was determined and displayed as a function of the fluorophore photoswitched (inset). The conventional steady-state anisotropy for each chimera was determined from the first data points of the photoswitching experiment. ANOVA indicated significant differences for all chimeras (*p*-value < 0.05). Cohen’s *d* values ranged 0.56–4.52. Data represent mean ± sem (*n* = 30, 33, 23, and 33 for D, D5D, D17D, and D32D, respectively). **d** The data points representing ~80% of the fluorescence were fitted to linear equations for experiments performed and analyzed as shown in (**c**). These were used to determine the anisotropy before and after photoswitching. The difference in anisotropy (delta r, black columns) is shown compared with its conversion to drFRET efficiency (white columns) using Eq. (2). Significant delta r differences were found for all chimeras (*p*-value < 0.05). Cohen’s *d* values ranged 0.67–7.66. Significant drFRET differences were found for all chimeras (*p*-value < 0.05). Cohen’s *d* values ranged 0.79–8.23. Data represent mean ± sem (*n* = 30, 33, 23, and 33 for D, D5D, D17D, and D32D, respectively). Circles overlaid on columns in the bar graphs represent individual data points. Source data are provided as a Source Data file.

To undergo homo-FRET, the same criteria for hetero-FRET must be met by fluorescent molecules^1,2^. For instance, they are subject to the inverse of the sixth power of the distance and thus must be located closely enough to energy transfer, usually <10 nm. The acceptor absorption and donor emission spectra must also overlap. While normally being less extensive than the spectral overlap found in many hetero-FRET experiments, most fluorophores have overlapping absorption and emission spectra. For example, the Dronpa^32^ photoswitchable fluorescent protein used extensively in this paper (Fig. 2a) as well as the commonly used donor, Cerulean^33^, and acceptor, Venus^34^ (Supplementary Fig. 3) show sufficient levels of spectral overlap to homo-FRET.

To test psAFRET as a viable method for measuring homo energy transfer, we followed previous approaches^8,23,35^ and developed tandem Dronpa dimers with a 5-amino acid linker (D5D), a 17-amino acid linker (D17D), and a 32-amino acid linker (D32D). In parallel, we also developed a Cerulean tandem dimer with a 5-amino acid linker (Cer-Cer) for photobleaching AFRET studies and utilized the Venus tandem dimers from the Vogel laboratory^8^ (Supplementary Fig. 3). We imaged Dronpa, Cerulean, or Venus through a Dual-View imager using a polarizing beamsplitter cube. This allowed us to image both parallel and perpendicular polarized light while Dronpa was being photoswitched off (Fig. 2b), or Cerulean and Venus were being photobleached (Supplementary Fig. 3). During photoswitching or photobleaching, both the parallel and perpendicular fluorescence emission signals decrease (Fig. 2b), but photoswitchable fluorescent proteins, such as Dronpa, allow the molecule to be photoswitched back on and the experiment repeated (Supplementary Fig. 4). Using the parallel and perpendicular channel intensities from the first images of the photoswitching series, we can calculate the mean steady-state anisotropies for each of the chimeras (Fig. 2c, inset). These show the decreases in anisotropy for the chimeras compared with Dronpa alone. Consistent with homo-FRET, the D5D shows the lowest level while the anisotropy increases with increasing linker length. On the other hand, the calculated anisotropy at each time point show approximately linear relationships when plotted as a function of the amount of fluorophore photoswitched off (Fig. 2c) or photobleached (Supplementary Fig. 3). Linear fits of these data provide a straightforward readout of the change in anisotropy (black columns, Fig. 2d). We note here that the anisotropy data are very noisy after ~0.8–0.9 of the Dronpa has been photoswitched off (Fig. 2c), and we use only the data points up to ~0.8 photoswitched for fitting. Using Eq. (2), the delta r determined for Dronpa, D5D, D17D, and D32D were converted into a drFRET efficiency (white columns, Fig. 2d). The chimeras with the longer linkers display smaller changes in anisotropy which lead to smaller drFRET efficiencies analogous to results obtained with Cerulean-Venus chimeras^35^ or the Dronpa-mCherry chimeras used in our previous study^23^. Important for the drFRET conversion, the psAFRET data allow anisotropy estimations under both FRET and non-FRET conditions, analogous to a hetero-FRET acceptor photobleaching experiment.

We also compared the delta r determined from psAFRET with delta r determined from conventional homo-FRET measurements (Supplementary Fig. 5). Using the Dronpa alone anisotropy as the non-FRET control condition, the delta r values determined for D17D and D32D are comparable, but the D5D psAFRET delta r is increased compared with the conventional measurement (Supplementary Fig. 5). We initially considered this to be an issue with the D5D psAFRET measurement, but conventional anisotropy measurements on Dronpa-5-mCherryAmber (D5ChA) suggested otherwise. The D5ChA is a chimera developed as a negative control in our previous psFRET study^23^. It has a mutation in mCherry which prohibits formation of a chromophore while still allowing protein formation. Since it cannot participate as an acceptor in FRET and is comparable in size to Dronpa, D5ChA can mimic the size of a D5D chimera while providing an estimation of the D5D anisotropy in the absence of homo-FRET. We find the steady-state anisotropy value for D5ChA to be slightly higher than Dronpa alone (Supplementary Fig. 5) suggesting that using Dronpa alone as our non-FRET control in conventional homo-FRET measurements may be underestimating the change in anisotropy for the D5D chimera. Of course, this should imply a discrepancy with the D17D and D32D delta r determinations since they are similar for both the psAFRET and conventional homo-FRET calculations. However, if we compare *r*_et0_ values, which is an estimation of the anisotropy in the absence of energy transfer produced by the psAFRET measurement, we find that the D5D *r*_et0_ is again slightly higher than the other chimeras (Supplementary Fig. 5) while the D17D and D32D *r*_et0_ values are similar to the Dronpa alone value.

### Photoswitching AFRET imaging of Dronpa tandem oligomers

**Fig. 3:**
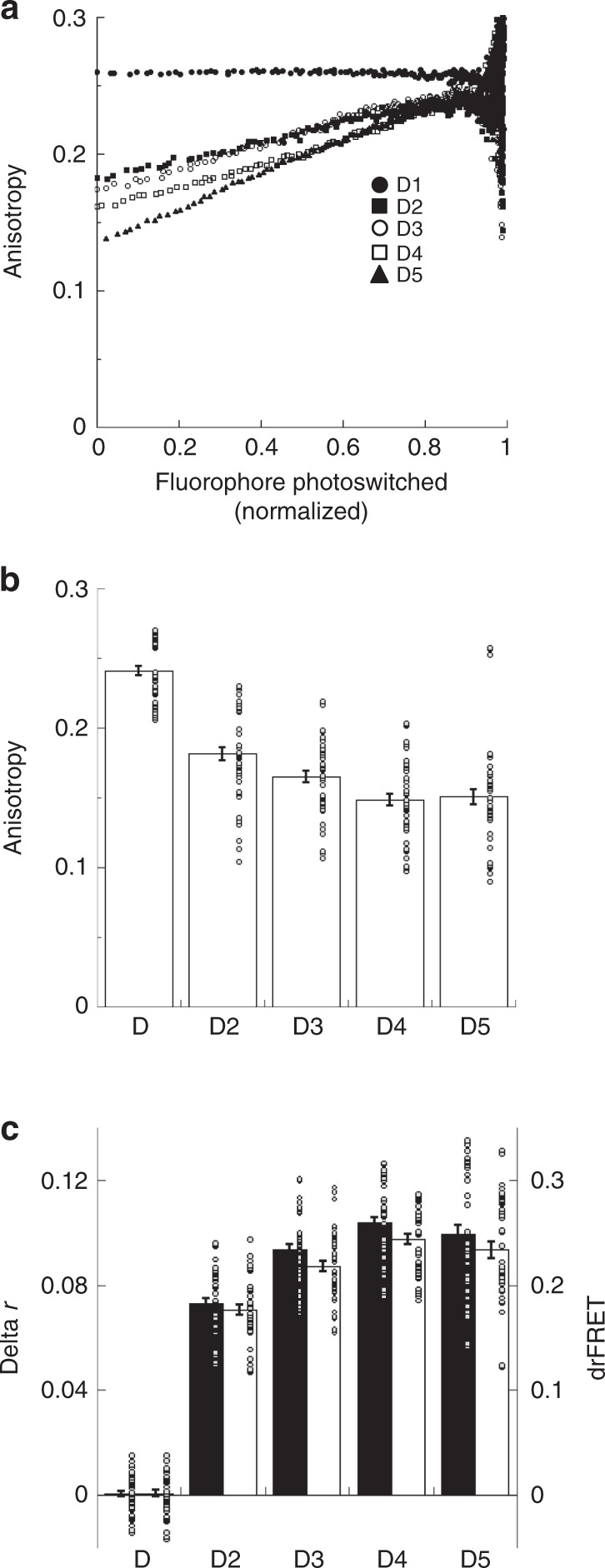
psAFRET measurements of Dronpa fluorescent protein oligomers. **a** COS-7 cells expressing Dronpa (black circles), D2 (black squares), D3 (white circles), D4 (white squares), or D5 (black triangles) were imaged and photoswitched. The anisotropy was determined and displayed as a function of the fluorophore photoswitched. **b** The conventional steady-state anisotropy for each chimera was determined from the first data points of the photoswitching experiment. ANOVA indicated significant differences for all comparisons except D3-D5 and D4-D5 (*p*-value < 0.05). Cohen’s *d* values ranged 0.54–3.12. Data represent mean ± sem (*n* = 45). **c** The data points representing ~80% of the fluorescence were fitted to linear equations for experiments performed and analyzed as shown in (**a**). These were used to determine the anisotropy before and after photoswitching. The difference in anisotropy (delta r, black columns) is shown compared with its conversion to drFRET efficiency (white columns) using Eq. (2). ANOVA indicated significant delta r differences for all comparisons except D3-D5 and D4-D5 (*p*-value < 0.05). Cohen’s *d* values ranged 0.62–6.44. ANOVA indicated significant drFRET differences for all comparisons except D3-D5 and D4-D5 (*p*-value < 0.05). Cohen’s *d* values ranged 0.7–6.74. Data represent mean ± sem (*n* = 45). Circles overlaid on columns in the bar graphs represent individual data points. Source data are provided as a Source Data file.

We again mimicked previous studies^8^ and followed these experiments by making chimeras containing multiple Dronpa molecules linked by 5-amino acid linkers. The anisotropy changes during photoswitching (Fig. 3a) as well as the decreases in conventional steady-state anisotropy measurements (Fig. 3b) with increasing numbers of Dronpa are again indicative of homo-FRET occurring in these chimeras. Linear fits to data in Fig. 3a up to ~ 0.8 fluorophore photoswitched are used to determine delta r (Fig. 3c) and these are converted into drFRET efficiencies using Eq. (2). The D2 chimera in these experiments is the same as D5D from Fig. 2 and give similar results. As the number of Dronpa molecules in the chimera increase, delta r and drFRET also increase (Fig. 3c). The increases in drFRET suggest that the Dronpa molecules in these multiple Dronpa chimeras are undergoing homo-FRET with more than one partner. Similar to our findings with D5D, we also find the psAFRET delta r values are slightly, but significantly, higher than conventional homo-FRET calculations (Supplementary Fig. 5).

Based on treatment of oligomer anisotropies as a function of fractional labeling or photobleaching, the psAFRET curves for D3-D5 are expected to show distinct upward curvatures^36^ instead of the linear profiles we observe. Previous^36^ as well as our own simulations (Supplementary Fig. 6) based on the binomial theorem clearly indicate these profiles are possible. Such distinctive photoswitching/photobleaching profiles are appealing since they could offer new approaches to studying protein oligomerization. However, we note that these distinctive curves assume a mean separation distance between the chromophores of ≤0.8 R_0_^11^. Our simulations and estimations for separation distances between the chromophores of the fluorescent proteins suggest this is unlikely for our test chimeras (Supplementary Fig. 6). Nevertheless, we proceeded to rule out a high illumination intensity as a trivial experimental parameter which might be masking the upward curvature. Our observations (Supplementary Fig. 7) are inconsistent with this explanation. To test if this could simply be a feature of the Dronpa molecule, we performed similar experiments by photobleaching Venus oligomers. While the V5 shows a slight curvature (Supplementary Fig. 3), it does not display the extreme behavior predicted by the binomial theorem (Supplementary Fig. 6), which suggests that the absence of those distinct profiles is not specific to Dronpa. While psAFRET data showing these characteristics may benefit future oligomerization studies, the stringent requirements for the fluorophore separation distances likely preclude testing and demonstration using our test chimeras.

### Photoswitching AFRET imaging under varied optical conditions

**Fig. 4:**
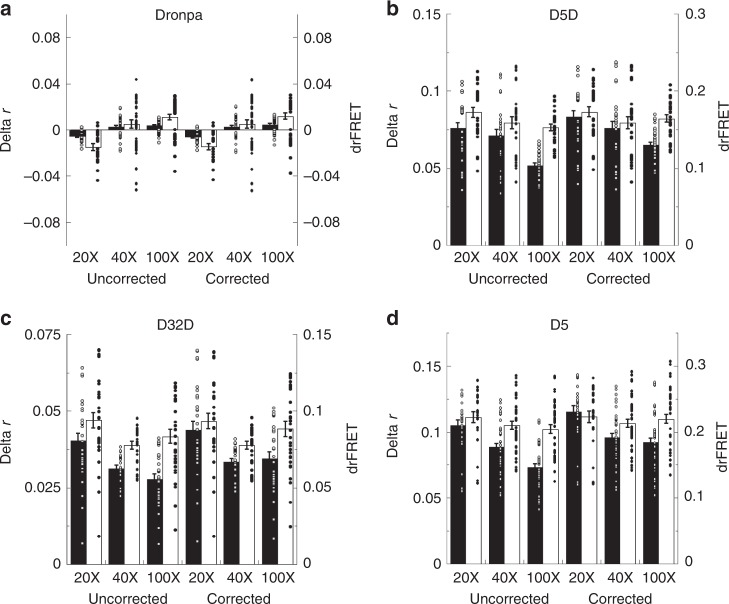
psAFRET measurements using low and high NA objective lenses. COS-7 cells expressing **a** Dronpa (*n* = 21, 39, and 39 for the 20X, 40X, and 100X, respectively), **b** D5D (*n* = 30 for each objective lens), **c** D32D (*n* = 30, 24, and 30 for the 20X, 40X, and 100X, respectively), or **d** D5 (*n* = 24, 42, and 45 for the 20X, 40X, and 100X, respectively) were imaged using either a 20X/0.75 NA, 40X/1.0 NA, or 100X/1.4 NA objective lens as indicated. The anisotropy during photoswitching was determined, plotted as a function of the fluorophore photoswitched, and the data points representing ~ 80% of the fluorescence were fitted to linear equations. These were used to determine the anisotropy before and after photoswitching. The difference in anisotropy (delta r, black columns) is shown compared with its conversion to drFRET efficiency (white columns) using Eq. (2). As indicated, data are uncorrected or corrected for use of high NA objective lenses using the Axelrod correction. Data represent mean ± sem (*n* = 21, 39, and 39 for 20X, 40X, and 100X, respectively, in (**a**); *n* = 30 for each in (**b**); *n* = 30, 24, and 30 for 20X, 40X, and 100X, respectively, in (**c**); *n* = 24, 42, and 45 for the 20X, 40X, and 100X, respectively, in (**d**)). ANOVA and Tukey–Kramer test results are listed in Supplementary Table 1. Circles overlaid on columns in the bar graphs represent individual data points. Source data are provided as a Source Data file.

The next set of experiments were aimed at demonstrating psAFRET capabilities in the context of high resolution imaging. Lenses with numerical aperture >1.0 are not commonly used in homo-FRET experiments since the steep collection angle lends to an inherent mixing of the polarizations. This lowers the measured anisotropy values which also translates into diminished measurements of homo-FRET induced changes in anisotropy^1^. For perspective, all of the experiments presented thus far were performed using a high numerical aperture 100X/1.4 NA objective lens. We repeated experiments on Dronpa, D5D, D32D, and Dronpa5(D5) using objective lenses with three different numerical apertures (Fig. 4). We find that the changes in anisotropy (Fig. 4, delta r, black columns) observed with the 100X/1.4 NA objective lens were indeed lower than those observed with a 40X/1.0 NA objective lens which were subsequently lower than those observed with a 20X/0.75 NA objective lens. We also compared uncorrected results with the same data after application of a correction for high NA objectives (corrected) derived by Axelrod^21^. The correction leads to increased *r*_et1_ and *r*_et0_ anisotropy values for all objective lenses but works better with the smaller objective numerical apertures (Supplementary Fig. 8). Moreover, we also find that the correction does not fully restore the delta r obtained with the 40X and 100X objective lenses to the same level as the 20X lens (Fig. 4, delta r, black columns). On the other hand, conversion of the anisotropy measurements to drFRET using Eq. (2) compensates well for polarization mixing with the higher NA objectives. For instance, the drFRET values are higher at the decreased anisotropy values for a given delta r (Supplementary Fig. 2). But more relevant to this experiment, given drFRET values produce decreased delta r values in conjunction with decreased anisotropy values under FRET and non-FRET conditions (Supplementary Fig. 2). As a consequence, the drFRET efficiency remains fairly constant for a given chimera whether determined from corrected or uncorrected data using all three objective lenses (Fig. 4, drFRET, white columns). Since the homo-FRET should be constant for a given chimera regardless of instrument parameters, this indicates that conversion of anisotropy changes to drFRET efficiency values will help improve the consistency of reported homo-FRET results.

### Photoswitching AFRET imaging of example oligomers

**Fig. 5:**
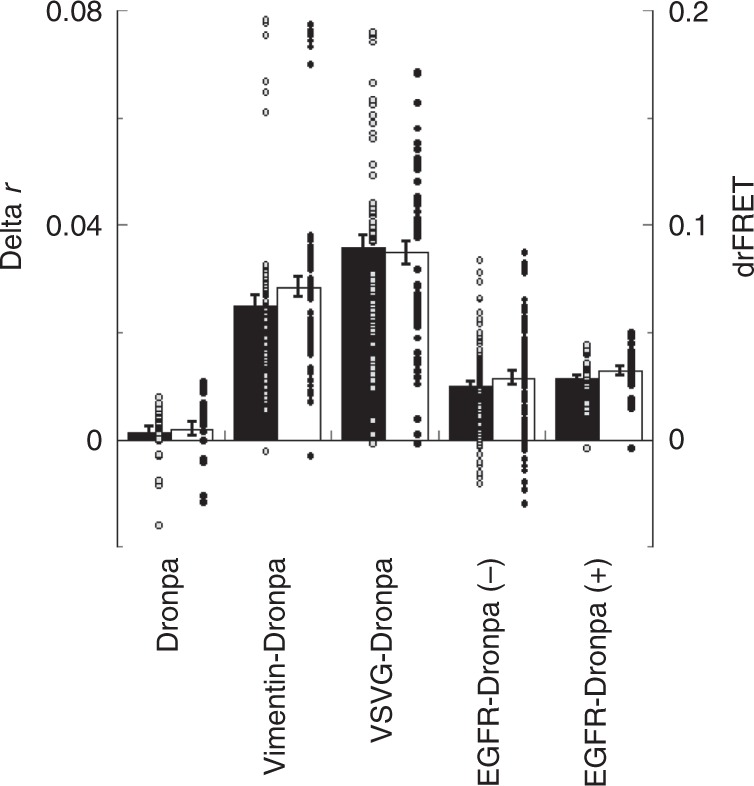
psAFRET measurements of Dronpa tagged proteins. COS-7 cells expressing Dronpa, Vimentin-Dronpa, VSVGtsO45-Dronpa, or EGFR-Dronpa were imaged and photoswitched. A subset of EGFR-Dronpa expressing cells were also treated with EGF. Dronpa fluorescence anisotropy was determined and plotted as a function of the fluorophore photoswitched. The data points representing ∼80% of the fluorescence were fitted to linear equations and used to determine the difference in anisotropy (delta r, black columns). These were also converted to drFRET efficiency (white columns) using Eq. (2). Data represent mean ± sem (*n* = 27, 72, 63, 108, and 30 for Dronpa, Vimentin-Dronpa, VSVGtsO45-Dronpa, EGFR-Dronpa (−EGF), and EGFR-Dronpa (+ EGF), respectively). Circles overlaid on columns in the bar graphs represent individual data points. Source data are provided as a Source Data file.

We further tested psAFRET by imaging Dronpa tagged molecules which are known to oligomerize (Fig. 5 and Supplementary Fig. 9). As a structural component of one class of intermediate filaments, vimentin forms dimers which associate to form tetramers which further oligomerize to form filaments of ~10 nm diameter^37^. Expression of vimentin-Dronpa in COS-7 cells followed by psAFRET experiments shows delta r values of ~ 0.025 which correspond to drFRET values of ~0.07. These data indicate that vimentin-Dronpa chimera molecules incorporate into intermediate filaments along with endogenous vimentin and are present in sufficient numbers to be located within homo-FRET distance. We also imaged the vesicular stomatitis virus G (VSVG) protein tsO45 variant tagged with Dronpa. The tsO45 variant is a temperature sensitive mutant of VSVG which is synthesized and inserted into the membrane of the endoplasmic reticulum (ER)^38^. When expressed at a non-permissive temperature of ~ 40 °C, VSVGtsO45 mis-folds and does not exit the ER, but at lower temperatures it becomes transport competent, exits the ER, trafficks through the Golgi apparatus en route to the plasma membrane^39^. Importantly for these experiments, biochemical^40^ and structural evidence^41^ indicate that the VSVG forms trimers in the plasma membrane. In agreement with the biochemical data, psAFRET experiments on VSVG-Dronpa show delta r values of ~0.036 and drFRET values of ~0.09, indicative of oligomerization. We imaged the epidermal growth factor receptor (EGFR) tagged with Dronpa expressed in COS-7 cells. The model for EGFR activation requires receptor dimerization^42^ so we considered this another straightforward test for our psAFRET method under cell imaging conditions. Similar to other FRET based studies in which the fluorophore is attached to the C-terminus^42^, we observed homo-FRET in both the absence (−) and presence (+) of excess EGF. Thus, psAFRET successfully reports on the self-interactions of several proteins known to oligomerize.

### Photoswitching AFRET of core histone interactions

**Fig. 6:**
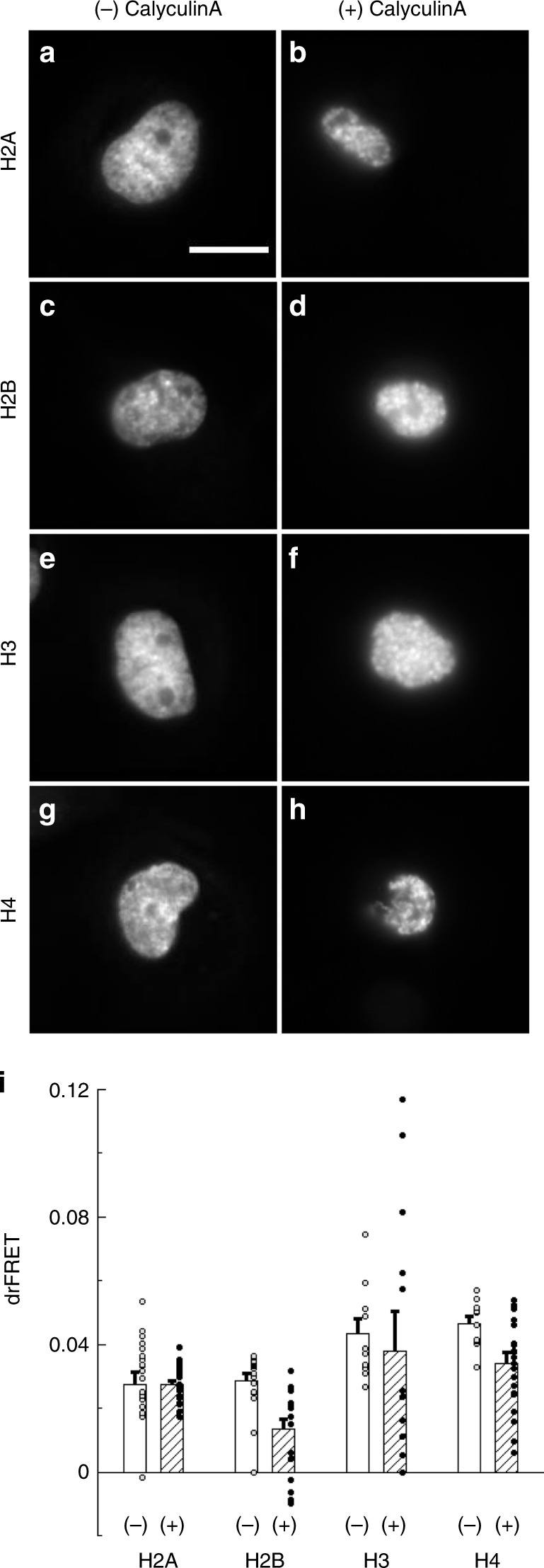
Chromatin compaction studies using psAFRET. COS-7 cells expressing **a**, **b** H2A-Dronpa, **c**, **d** H2B-Dronpa, **e**, **f** H3-Dronpa, and **g**, **h** H4-Dronpa at similar levels were imaged and photoswitched. The cells were (**a**, **c**, **e**, **g**) mock treated or (**b**, **d**, **f**, **h**) treated with 100 nM Calyculin A for 1 h before imaging. Scale bar in (**a**) is 10 µm and applies to all images. The anisotropy during photoswitching was determined, plotted as a function of the fluorophore photoswitched, and (**i**) fitted to linear equations to determine the change in anisotropy. The values from mock (white columns) and Calyculin A (hatched columns) treated experiments were converted to drFRET efficiency using Eq. (2). Data represent mean ± sem (*n* = 19, 20, 20, 10, 13, 10, and 20 for H2A−, H2A +, H2B−, H2B +, H3−, H3 +, H4−, H4 +, respectively). Two-tailed *t*-tests indicated a difference (*p*-value < 0.01) in the mean drFRET values of control versus Calyculin A treated cells for H2B-Dronpa and H4-Dronpa. Cohen’s *d* values were found to be 1.4 and 1.1, respectively. Circles overlaid on columns in the bar graphs represent individual data points. Source data are provided as a Source Data file.

Our final psAFRET example takes advantage of the predicted locations of the C-terminal tails of core histone proteins within the nucleosome. Based on nucleosome structures, we reasoned that tagging Dronpa to each of the core histone C termini would preferentially place the fluorescent protein at several different positions (Supplementary Fig. 10) and provide readouts for nucleosome interactions during chromatin condensation. We transiently expressed each of the core histone proteins, H2A (Fig. 6a, b), H2B (Fig. 6c, d), H3 (Fig. 6e, f), and H4 (Fig. 6g, h) tagged with Dronpa and imaged using our psAFRET method in absence (−) and presence (+) of Calyculin A treatment. Calyculin A is a type 1 and type 2A protein phosphatase inhibitor and treatment of cells results in DNA condensation outside the normal mitotic cycle^43^. In the absence of Calyculin A (Fig. 6a, c, e, g), the tagged histones show nuclear distributions and small but observable levels of homo-FRET (Fig. 6i, white columns). Incubation with Calyculin A (Fig. 6b, d, f, h) results in condensation of the DNA into structures qualitatively similar to those observed in mitotic prophase. However, the homo-FRET signals observed for each tagged histone differ. For instance, H2A-Dronpa and H3-Dronpa homo-FRET signals show little change, and H4-Dronpa and H2B-Dronpa show reductions in drFRET. These differences do not appear to be due to large differences in expression levels, since we compare cells with similar fluorescence signals (Supplementary Fig. 11). Thus, the varying homo-FRET responses observed with the histone-Dronpa chimeras indicate that the psAFRET method may assist in providing new insight into large scale chromatin rearrangements.

## Discussion

Since protein oligomerization has roles in a number of cell processes, the capability to monitor these interactions with straightforward, consistent, and accurate methods facilitates a better understanding of the proteins’ behavior. Homo-FRET between similar fluorophores has the advantage of requiring only a single fluorescence channel to monitor protein oligomerization, yet it has the disadvantage in that monitoring homo-FRET requires the capability to monitor changes in fluorescence anisotropy. Here, we have introduced a new variation on imaging energy transfer between similar proteins using changes in the anisotropy of the fluorescence emission of photoswitchable fluorescent proteins as they are photoswitched to the off state. Just as with previous approaches relying on the photobleaching of conventional fluorophores^6,16^, this technique is designed to provide anisotropy values under FRETing and nonFRETing conditions for the same labeled cellular protein sample. Photoswitching, on the other hand, provides distinct advantages over photobleaching including the capability to turn off the fluorescence more quickly with less illumination intensity as well as the capability to photoswitch the protein back on and repeat the experiment^23^.

We have tested and demonstrated psAFRET with several Dronpa chimeras and found the change in anisotropy to be indicative of homo-FRET. Moreover, we noted that changes in anisotropy during photoswitching showed a linear relationship to the amount of Dronpa fluorescence which was photoswitched off. We found this to be a useful characteristic of the data since we could fit the psAFRET photoswitching curves to linear equations and more easily estimate delta r by extrapolating to zero fluorescence intensity. By doing so, we could estimate the anisotropy of the chimera or tagged protein in both the presence (*r*_et1_) and the absence (*r*_et0_) of homo-FRET, which is analogous to the acceptor photobleaching technique commonly utilized in hetero-FRET experiments.

Although equations converting anisotropy values into FRET efficiencies are available in the literature^27–29^, we were uncertain that assumptions made for those equations would be valid for our data using photoswitchable fluorescent proteins. Therefore, the unique capability of a psAFRET experiment to provide *r*_et1_ and *r*_et0_ values for each sample prompted us to derive a separate equation to convert these two values into drFRET, a value akin to but distinct from a hetero-FRET efficiency.

The drFRET value simply represents the percentage increase in fluorescence signal in the perpendicular channel compared with the total signal. What is the relevance for this conversion to a FRET-like efficiency? Our observations with three different objective lenses show that converting the observed anisotropy changes in psAFRET measurements to drFRET more faithfully reports the homo-FRET occurring in our Dronpa chimeras even under conditions where polarization mixing is occurring. Thus, this value can standardize the same homo-FRET measurements made across varied optical configurations.

We include several examples of psAFRET use with known oligomeric proteins including vimentin, EGFR, histones, and VSVG and were able to measure homo-FRET from each of these chimeras. One surprising result came from the chromatin condensation studies using tagged core histones. Based on the structure of the nucleosome and the positioning of the C-terminal tails of histone proteins, we reasoned that the Dronpa tag should be located at distinct points on the periphery of the nucleosome. We further reasoned that homo-FRET measurements might provide a readout for structural alterations during chromatin condensation. We considered that the signal could arise from tagged histones located within the same nucleosome, which would likely show little change due to chromatin condensation. Homo-FRET could also occur between neighboring nucleosomes which are organized into a 10 nm fiber and then into higher order chromatin fibers, such as the 30 nm fiber^44^ or a recently proposed 24 nm fiber^45^. However, an alternate model suggests an absence of a 30 nm fiber under physiological conditions and that chromatin exists mainly in a disordered 10 nm fiber state^46^. Finally, we also considered that homo-FRET could be occurring due to long range chromatin interactions arising from even higher order folding of the proposed fibers, whether they are 10, 24, or 30 nm in size.

With perhaps the exception of H2A-Dronpa, our most simple expectation was that all histone chimeras would show increased homo-FRET after condensation since the volume encompassed by the chromatin would be decreased due to compaction. Indeed, H2A-Dronpa showed little change in homo-FRET, but we also observed little change for H3-Dronpa. More surprisingly, we observed reduced homo-FRET signals for H2B-Dronpa and H4-Dronpa after compaction. Based on several proposed models for the 30 nm fiber^44^, we predicted the Dronpa tag on H2A to be located in the center of the fiber (Supplementary Fig. 10). The close proximity (< 10 nm) within the center would likely lead to H2A-Dronpa homo-FRET interactions, which we observed, and those interactions would be unlikely to change during compaction, which we also observed. We note that while these observations would be expected for the 30 nm fiber models, they are not exclusive of the other proposed models. However, the H2B-Dronpa predicted location is on the periphery of a 30 nm fiber (Supplementary Fig. 10) and our expectation was that homo-FRET would increase, rather than decrease, in response to condensation as the fibers packed closer together. Moreover, our expectations of homo-FRET increases regardless of the chromatin structure model, led to our simplistic expectations of chromatin condensation. Our psAFRET method may currently be limited by the lack of FRET efficiency calculations and subsequent separation distance estimates, but it does provide a straightforward method to test some of the more straightforward predictions of popular chromatin structure models during condensation.

In summary, drFRET values determined from psAFRET experiments provide a straightforward way to monitor and report homo-FRET with consistency across laboratories and imaging platforms. The anisotropy values necessary to calculate drFRET are determined from each sample, which circumvents comparisons of FRET experiments with non-FRET controls to determine the anisotropy changes. Thus, homo-FRET estimations on obligate oligomers can also be made in a reliable manner. The psAFRET approach does not provide as much information as time-resolved approaches, but it also does not require access to highly specialized instrumentation, expertise in the use of that instrumentation, nor the experience for proper data analysis. Thus, the simple nature of this approach makes it readily accessible to biologists with modest experience in conventional imaging techniques to study oligomerization of their proteins of interest.

## Methods

### Cell culture

Cell culture was performed as reported previously^23^ and reiterated here. COS-7 cells were obtained from ATCC (Manassas, VA, USA, catalog number CRL-1651) and maintained in standard DMEM-HG medium supplemented with 10% (vol/vol) heat-inactivated FBS, 1 mM sodium pyruvate and 2 mM Glutamax in a 5% CO_2_ incubator at 37 ^°^C. For imaging, the cells were plated in Bioptechs Delta-T dishes (Bioptechs, Butler, PA, USA, product number 04200417B) and grown to ~ 70% confluency. Transient transfections were done with various plasmids according to manufacturer’s instructions using 1–2 μg DNA and 3ul of X-tremeGene HP DNA transfection reagent (Roche, Manheim, Germany, product number 06366236001). The transfected cells were incubated at 37 °C and 5% CO_2_ incubator for 24–48 h before imaging. The cell culture reagents DMEM-HG medium (catalog number 11960), fetal bovine serum (FBS, catalog number 10082), sodium pyruvate (catalog number 11360) and Glutamax (catalog number 35050) were obtained from Invitrogen (Life Technologies, Grand Island, NY, USA). Calyculin A was obtained from Sigma (catalog number C 5552).

### Construction of recombinant DNA plasmids

Oligonucleotide primers were synthesized by Eurofins Genomics (Louisville, KY). Polymerase chain reactions (PCR) were performed with Phusion (New England Biolabs, Ipswich, MA) or Pfu Turbo (Stratagene). Restriction enzymes were from New England Biolabs. Digested fragments were gel purified using QIAquick Gel Extraction Kit (Qiagen, Germantown, MD). Ligation reactions were performed with T4 DNA Ligase from Invitrogen (Life Technologies, Grand Island, NY) or New England Biolabs, Inc. All newly constructed plasmids had sequences verified by Eurofins Genomics (Louisville, KY).

The mCerulean-mCerulean (Cer-Cer) plasmid was constructed by PCR amplifying mCerulean (from mCeruelan-C1) using the N-terminal annealing primer 5′-GATCAGATCTGTGAGCAAGGGCGAGGAG-3′ containing a BglII site (underlined) and the C-terminal annealing primer 5′-GATCGAATTCCTTGTACAGCTCGTCCAT-3′ containing an EcoRI site (underlined). The mCerulean PCR product and mCerulean-C1 were digested with BglII and EcoRI then ligated together creating the five-amino acid linker SGLRS. The Venus oligomer plasmids^8^ were purchased from Addgene (Watertown, MA; catalog numbers 29423, 27814, 29425, 29426, and 27813).

The Dronpa-5-Dronpa (D5D or D2) plasmid was constructed by PCR amplifying Dronpa (from pDronpa-C1) without its first methionine (ATG) using the N-terminal annealing primer 5′-GATCAGATCTAGTGTGATTAAACCAGAC-3′ containing a BglII site (underlined) and the C-terminal annealing primer 5′-GATCGAATTCCTTGGCCTGCCTCGGCAG-3′ containing an EcoRI site (underlined). Both the Dronpa PCR product and pDronpa-C1 were digested with BglII and EcoRI then ligated together creating the five-amino acid linker SGLRS.

The Dronpa-17-Dronpa (D17D) plasmid was constructed by PCR amplifying Dronpa (from Dronpa-N1) using the N-terminal primer 5′-GATCGGTACCATGAGTGTGATTAAACCAG-3′ containing a KpnI site (underlined) and the C-terminal primer 5′-GATCGGATCCTTACTTGGCCTGCCTCGG-3′ containing a BamHI site (underlined). The Dronpa PCR product was digested with KpnI and BamHI then ligated into a similarly digested Dronpa-17-mCherry^23^ to maintain the 17-amino acid linker, SGLRSRAQASNSAVDGT.

The Dronpa-32-Dronpa (D32D) plasmid was constructed by digesting pDronpa-N1 with AgeI and NotI to isolate a fragment containing the cDNA for Dronpa. This was ligated into a similarly digested Dronpa-32-mCherry^23^ to maintain the thirty two-amino acid linker, TSGLETRDIRSENLYFQGPREFPGGTAGPVAT.

The Dronpa-Dronpa-Dronpa (D3) plasmid was constructed by PCR amplifying Dronpa (from pDronpa-C1) without its first methionine (ATG) using the N-terminal annealing primer 5′-GATCGTCGACGGGTGAGCAAGGGCGAGGAG-3′ containing a SalI Site (underlined) and the C-terminal annealing primer 5′-GATCGGATCCCTTGTACAGCTCGTCCAT-3′ containing a BamHI site (underlined). Both the Dronpa PCR product and pDD (described above) were digested with SalI and BamHI then ligated together creating the six-amino acid linker EFCSRR between the middle Dronpa and the C-terminal Dronpa.

The Dronpa-Dronpa-Dronpa-Dronpa (D4) plasmid was constructed by PCR amplifying Dronpa (from Dronpa-N1) using the N-terminal annealing primer 5′-GATCGGATCCACTGGAACTAGTGTGATTAAACCAG-3′ containing a BamHI site (underlined) and the C-terminal annealing primer 5′-GATCTCTAGACTTGGCCTGCCTCGGC-3′ containing a XbaI site (underlined). The Dronpa PCR product was digested with BamHI and XbaI then ligated into a similarly digested D3-C1 plasmid to add a Dronpa to the end of the D3 with a GSTGT (five-amino acid linker).

The Dronpa-Dronpa-Dronpa-Dronpa-Dronpa (D5) plasmid was constructed by PCR amplifying Dronpa (from Dronpa-N1) using the N-terminal annealing primer 5′-GATCTCCGGAACTGGAACTAGTGTGATTAAACCAG-3′ containing a BspEI site (underlined) and the C-terminal annealing primer 5′-GATCAGATCTAGTTCCAGTCTTGGCCTGCCTCGGC-3′ containing a BglII site (underlined). The Dronpa PCR product and D4-C1 plasmid were digested with BspEI and BglII then ligated together to place a Dronpa at position 2 within the D4 chimera. This resulted in a five-amino acid linker (SGTGT) between the first position Dronpa and the second position Dronpa and a five-amino acid linker (TGTRS) between the second position Dronpa and the third position Dronpa.

As previously described^23^, a plasmid encoding Vimentin-PSmOrange^47^ was the gift of Vlad Verkhusha (Albert Einstein College of Medicine). It was digested with NheI and BamHI to isolate a fragment containing the cDNA for vimentin. This was ligated into a similarly digested Dronpa-N1 plasmid to produce Vimentin-Dronpa. A plasmid encoding EGFR-PSmOrange^47^ was the gift of Vlad Verkhusha (Albert Einstein College of Medicine). It was digested with AgeI and NotI to isolate a fragment containing the cDNA for EGFR. This was ligated into a similarly digested Dronpa-N1 plasmid to produce EGFR-Dronpa. The VSVGtsO45-NL-Dronpa was constructed by digesting a previously described VSVGtsO45-NL-EGFP^48^ with XhoI and EcoRI to isolate VSVGtsO45-NL which was ligated into a similarly digested Dronpa-N1 plasmid. The H2B-Dronpa chimera construction was previously described^23^ and the construction of the Dronpa-N1 plasmid was described previously^49^. The H2A-Dronpa, H3-Dronpa, and H4-Dronpa plasmids were gifts from Michael Davidson (Florida State University).

### Microscopy

The microscope used for anisotropy experiments is a home built system on a Nikon TE2000 base reported previously^23^ and described here with necessary alterations. The objective lenses used for imaging were a Nikon 20X/0.75 NA Plan Apo, a Nikon 40X/1.0 NA Oil Plan Apo and a Nikon 100X/1.4 NA Oil Plan Apo. A 405 nm laser (LaserBoxx, Oxxius, Lannion, France) was used to photoswitch proteins to the on state and a 488 nm laser (Sapphire, Coherent Inc., Santa Clara, CA, USA) was used to image Dronpa and mVenus. A 442 nm laser (LaserBoxx, Oxxius, Lannion, France) was used to excite and photobleach mCerulean. Laser lines were combined using appropriate dichroic mirrors. The 405 nm laser current was controlled using the ESIo AOTF controller (ESImaging, Folkestone, Kent, UK) and was shuttered using a diaphragm shutter with controller (part# SH025T, Thorlabs, Inc., Newton, NJ) triggered using the ESIo AOTF controller (ESImaging). All other laser lines were controlled using an AOTF (Gooch & Housego PLC, Ilminster, UK). All lasers are passed through a linear polarizer (part # WP25M-VIS, Thorlabs) and directed toward the objective using either a quad band dichroic (part # Di03-R405/488/561/635, Semrock, Rochester, NY), a 488 nm dichroic (part # DiO3-R488, Semrock, Rochester, NY) or a CFP dichroic (part # FF458-Di03-25x36 Semrock, Rochester, NY) mirror. Samples are illuminated in epi-illumination mode and emission is collected using same objective. The emission is passed through the dichroic and reflected toward camera using 45-degree mirror. Emission is passed through appropriate emission filters and passed through a Dual-View splitter (Photometrics, Tucson, AZ). A DV2 POL cube (part # DV2-POL-CUBE-KIT, Photometrics) was inserted into the Dual-View imager which splits emission in orthogonal polarizations allowing a simultaneous recording of both images using a PCO Edge 4.2 LT (PCO AG, Kelheim, Germany) camera. The microscope was controlled using MicroManager^50^. Unless otherwise specified, estimated power densities ranged from ~0.03 to ~0.2 W cm^−2^ for 488 nm photoswitching.

### Image analysis

Acquired images were analyzed using a macro written in Fiji^51,52^. The macro performs the following steps in the sequence mentioned. First, a user defined region of interest (ROI) in the parallel image and a corresponding region in perpendicular image is selected. The mean intensity from the ROI for both parallel and perpendicular images are determined at each time point. Offset and background signals are subtracted using data from sample regions containing no cells. For some analyses, the total fluorescence (*F*_tot_) was calculated using $F_{{\mathrm{tot}}} = I_{||} + 2gI_ \bot$, the values were normalized to the time point at the start of the photoswitching cycle, and then fitted with a single exponential with offset equation *y* = a*e^(−*bx*)^ + *c* using the ImageJ curve fitting function, where *y* is the fluorescence at time point *x*, *a* is the fluorescence at time 0, *b* is the rate constant, and *c* is the offset. For uncorrected anisotropy analyses, the average anisotropy (*r*) of the ROI was calculated at each time point using $r = \frac{{I_{||} - gI_ \bot }}{{I_{||} + 2gI_ \bot }}$ where *g* represents a correction factor to accommodate any polarization bias in the optical pathway. We estimate the correction factor by collecting images focusing into a solution of fluorescein isothiocyanate (FITC) (part# F-7250, Sigma, St. Louis, MO). For anisotropy analyses corrected for use of high numerical aperture objective lenses, we used the approach detailed by Axelrod^21,22^ using $r = \frac{{I_{\mathrm{z}} - I_{\mathrm{y}}}}{{I_{\mathrm{z}} + 2I_{\mathrm{y}}}}$. If the fluorophores are randomly oriented, *I*_x_ = *I*_y_, and the following Eqs. (3) and (4)

3 $$I_{||} = K_{\mathrm{c}}I_{\mathrm{z}} + K_{\mathrm{b}}I_{\mathrm{y}} + K_{\mathrm{a}}I_{\mathrm{x}}$$

4 $$gI_ \bot = K_{\mathrm{b}}I_{\mathrm{z}} + K_{\mathrm{c}}I_{\mathrm{y}} + K_{\mathrm{a}}I_{\mathrm{x}}$$

can be rearranged to solve for the variables, *I*_*z*_ and *I*_*y*_.

5 $$I_{\mathrm{y}} = \frac{{I_{||}K_{\mathrm{b}} - gI_ \bot K_{\mathrm{c}}}}{{K_{\mathrm{b}}K_{\mathrm{b}} + K_{\mathrm{a}}K_{\mathrm{b}} - K_{\mathrm{c}}K_{\mathrm{c}} - K_{\mathrm{a}}K_{\mathrm{c}}}}$$

6 $$I_{\mathrm{z}} = \frac{{gI_ \bot K_{\mathrm{b}} + gI_ \bot K_{\mathrm{a}} - I_{||}K_{\mathrm{c}} - I_{||}K_{\mathrm{a}}}}{{K_{\mathrm{b}}K_{\mathrm{b}} + K_{\mathrm{a}}K_{\mathrm{b}} - K_{\mathrm{c}}K_{\mathrm{c}} - K_{\mathrm{a}}K_{\mathrm{c}}}}$$

The variables *K*_a_, *K*_b_, and *K*_c_ are defined by the following Eqs. (7), (8), and (9).

7 $$K_{\mathrm{a}} = \frac{{2 - 3{\mathrm{cos}}\theta + {\mathrm{cos}}^3\theta }}{3}$$

8 $$K_{\mathrm{b}} = \frac{{1 - 3{\mathrm{cos}}\theta + 3{\mathrm{cos}}^2\theta - {\mathrm{cos}}^3\theta }}{{12}}$$

9 $$K_{\mathrm{c}} = \frac{{5 - 3{\mathrm{cos}}\theta - {\mathrm{cos}}^2\theta - {\mathrm{cos}}^3\theta }}{4}$$

The variable *θ* represents the collection angle of the lens determined from ${\mathrm{NA}} = n \ast {\mathrm{sin}}\theta$ where *n* represents the index of refraction and NA is the numerical aperture of the objective lens.

The anisotropy values were then plotted as a function of the fluorophore photoswitched or photobleached. Fluorophore photoswitched or photobleached was determined by $F\left( t \right) = \frac{{I\left( {t_0} \right) - I\left( t \right)}}{{I\left( {t_0} \right)}}$ where *I*(*t*_0_) is the total fluorescence at the initial time point and *I*(*t*) is the total fluorescence at time point *t*. Data up to ~0.8 fluorophore photoswitched was fitted to a linear equation *y* = *a* + b*x using the ImageJ curve fitting function to determine the slope of the line, where *y* = *r*, *x* = 1−*F*(*t*), and *b* is the slope of the line. The slope of the line was used to extrapolate to complete fluorophore photoswitching and determine the change in anisotropy due to homo-FRET. The estimated standard error of the regression model was determined by ${\it{s}} = \sqrt {\frac{{{\sum} {\left( {{\it{r}}_{\mathrm{t}} - {{\hat r}}_{\it{t}}} \right)^2} }}{{n - 2}}}$ where *r*_t_ is the anisotropy at time point *t*, $\hat r_{\mathrm{t}}$ is the fitted anisotropy at time point *t*, and *n* is the number of points used in the linear regression. The error in determination of the slope $( {s_{{\hat{\upbeta}}_1}} )$ was calculated by $s_{{\hat{\upbeta}}_1} = \frac{s}{{\sqrt {{\mathrm{SS}}_{{\mathrm{xx}}}} }}$, where ${\mathrm{SS}}_{{\mathrm{xx}}} = {\sum} {x_{\mathrm{t}}^2} - \frac{{\left( {{\sum} {x_t} } \right)^2}}{n}$ and *x*_t_ is the fluorescence photoswitched at time point *t*. Typical errors in anisotropy slope, $s_{{\hat{\upbeta}}_1}$, determinations, were <0.001. For multiple comparisons, ANOVA tests were performed using a function in Microsoft Excel. When significant differences between groups were found, Tukey–Kramer tests were performed. Where appropriate, two-tailed *t*-tests were performed using a function in Microsoft Excel assuming unequal variances. Cohen’s *d* was calculated by ${\it{d}} = \frac{{\sqrt {\left( {\bar x_{{\mathrm{con}}} - \bar x_{{\mathrm{exp}}}} \right)^2} }}{{s_{\mathrm{p}}^2}}$, where $\bar x_{{\mathrm{con}}}$ is the mean for the control values, $\bar x_{{\mathrm{exp}}}$ is the mean for the experimental values, and $s_{\mathrm{p}}^2$ is the pooled variance for the two datasets.

### Reporting summary

Further information on research design is available in the Nature Research Reporting Summary linked to this article.

## Supplementary information

Supplementary Information

Reporting Summary

## Data Availability

The data supporting the findings of this study are available upon reasonable request from the corresponding author. Source data for Figs. 2, 3, 4, 5e, and 6i are also provided as a Source Data file.

## Source data

Source Data

## Code availability

The ImageJ macros used to automate the image analyses will be made available upon request from the corresponding author. It is also available at http://sites.imagej.net/Pattersg/.
