# Supplementary material for: Imaging of fluorescence anisotropy during photoswitching provides a simple readout for protein self-association
Source: Nat Commun. 2020 Jan 7;11:21. doi: 10.1038/s41467-019-13843-6 (PMC6946710; doi:10.1038/s41467-019-13843-6)
Supplement: Supplementary file 1 — Supplementary Information [file 41467_2019_13843_MOESM1_ESM.pdf]

Supplementary Information

Imaging of fluorescence anisotropy during photoswitching provides a simple readout for protein self-association

Ojha et al.

Supplementary Note 1

Here, we consider homo-FRET between fluorescent proteins, which are known to have a high intrinsic anisotropy due to their relatively slow rotational correlation times. Energy transfer between fluorescent proteins leads to polarization scrambling and decreased anisotropy. We approached this problem by describing homo-FRET as a sensitized emission experiment with the parallel and perpendicular emission channels being analogous to the donor and FRET emission channels, respectively. We include all steps in our approach to this problem which will allow readers to follow our reasoning and check our work. To begin, consider the coordinates associated with an anisotropy experiment.

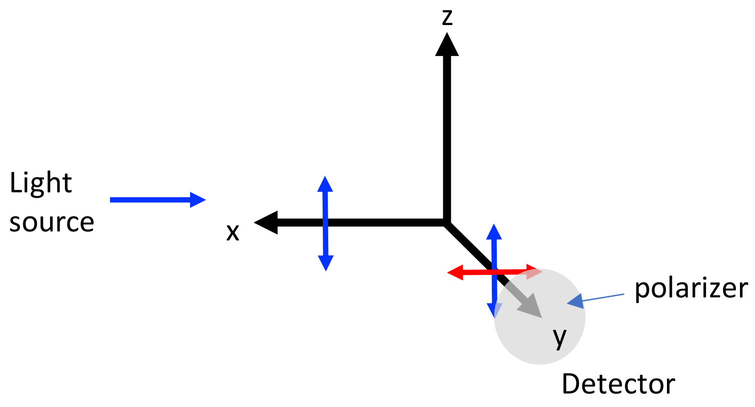

In a typical spectrometer based experiment, excitation light polarized in the z direction is transmitted along the x axis and excites a sample. The emission is detected in one or both directions along the y axis. Emission is collected through polarization filters which transmit light polarized parallel with the z axis (blue arrow in the figure) or polarized parallel with the x axis (perpendicular to z, red arrow in the figure). The intensity difference between I_z and I_x polarized light indicates the polarization of the sample. Polarization can provide a readout of the fluorophore's rotational motion which may report on its microenvironment or molecular binding events. Polarization (P) is defined by

$$P = \frac{I_z - I_x}{I_z + I_x} \tag{1}$$

where I_z and I_x represent light polarized parallel with the z axis (parallel with the excitation light polarization) or polarized parallel with the x axis (perpendicular to the excitation light polarization).

In keeping with our sensitized emission analogy, I_z represents the donor channel and I_x represents the FRET channel. Similar to sensitized emission, I_x will contain the energy transfer signal (ET) and crosstalk from non-FRETing donor molecules as well as direct excitation and emission for this orientation.

$$I_x = \mathrm{ET} + \ \mathrm{crosstalk} \tag{2}$$

For polarization measurements on a population of molecules in the absence of homo-FRET, any given P and I_z values will produce an expected I_x. Here, we consider this signal to be the non-FRETing crosstalk component. Therefore, measurements on these control samples (designated $_{et0}$) will provide the necessary control ($I_{x\ et0}/I_{z\ et0}$) ratio to determine the non-FRETing crosstalk ratio. In a population of molecules undergoing homo-FRET, the ratio $I_{x\ et1}/I_{z\ et1}$ ($_{et1}$ is used to designate an experimental sample undergoing energy transfer) will differ due to increased perpendicular polarized light and decreased parallel polarized light. However, for molecules in this population not undergoing homo-FRET, the ratio between the measured $I_{z\ et1}$ value and a corresponding crosstalk component ($I_{x\ ct}$) will be the same. Here, the subscript $_{ct}$ is used to designate the crosstalk signal. Therefore, for the non-FRETing population,

$$\frac{I_{\mathrm{x\ ct}}}{I_{\mathrm{z\ et1}}} = \frac{I_{\mathrm{x\ et0}}}{I_{\mathrm{z\ et0}}} \tag{3}$$

which allows the non-FRETing contribution in $I_{x\ ct}$ to be determined by the following.

$$I_{\mathrm{x\ ct}} = I_{\mathrm{z\ et1}} \frac{I_{\mathrm{x\ et0}}}{I_{\mathrm{z\ et0}}} \tag{4}$$

In keeping with the sensitized emission experiment analogy, $\frac{I_{\mathrm{x\ et0}}}{I_{\mathrm{z\ et0}}}$ is equivalent to the bleedthrough correction factor which is required to remove donor crosstalk from the FRET channel. Next, we substitute our newly defined terms, the measured I_x value ($I_{x\ et1}$), the corrected energy transfer contribution ($ET_{x\ c}$) for FRET, and $I_{x\ ct}$ for the crosstalk contribution in Supplementary Equation 2.

$$I_{\mathrm{x\ et1}} = \mathrm{ET}_{\mathrm{x\ c}} + I_{\mathrm{x\ ct}} \tag{5}$$

Substitute for $I_{x\ ct}$ using Supplementary Equation 4, rearrange, and this allows the sensitized emission due to homo-FRET to be determined by

$$\mathrm{ET}_{\mathrm{x\ c}} = I_{\mathrm{x\ et1}} - I_{\mathrm{z\ et1}} \frac{I_{\mathrm{x\ et0}}}{I_{\mathrm{z\ et0}}} \tag{6}$$

Important to note here is that $ET_{x\ c}$ does not represent all of the fluorescence lost from $I_{z\ et1}$ due to homo-FRET. The light polarized parallel with the y axis (I_y) will not be detected with the configuration just described and it is necessary to account for the total isotropic fluorescence ($I_x + I_y + I_z$) in an anisotropy (r) experiment. In addition, just as in a sensitized emission experiment, proper calculation of energy transfer efficiency requires determination of the intensity of the donor in the absence of homo-FRET.

Before proceeding with the derivation based on spectrometer configurations, we instead consider both excitation and detection of light along the x-axis which is the configuration for an epifluorescence microscope. Here, we would collect z-polarized and y-polarized light and similar equations could be derived if we simply substitute I_y for I_x.

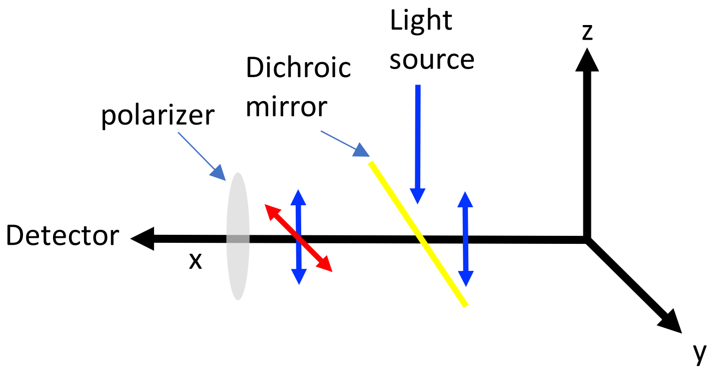

$$P = \frac{I_z - I_y}{I_z + I_y} \tag{7}$$

$$ET_{y\,c} = I_{y\,et1} - I_{z\,et1}\frac{I_{y\,et0}}{I_{z\,et0}} \tag{8}$$

Thus, to account for the decrease in I_z fluorescence due to homo-FRET and find the total sensitized emission signal (ET_c), we must add the values of $ET_{x\,c}$ and $ET_{y\,c}$.

$$ET_c = ET_{x\,c} + ET_{y\,c} \tag{9}$$

However, in most polarized imaging or spectroscopy experiments, we do not collect data along both x and y axes and only have one perpendicular channel. This simplifies by noting that if we assume the population of molecules is randomly oriented, then x-polarized light can be considered equal to that of y-polarized light. Therefore, $I_x = I_y$, and the total isotropic fluorescence ($I_x + I_y + I_z$) can be described by $2I_y + I_z$. Similarly, ET_c can be determined by substituting Supplementary Equations 6 and 8 into Supplementary Equation 9

$$ET_c = \left(I_{x\,et1} - I_{z\,et1}\frac{I_{x\,et0}}{I_{z\,et0}}\right) + \left(I_{y\,et1} - I_{z\,et1}\frac{I_{y\,et0}}{I_{z\,et0}}\right) \tag{10}$$

and then substituting I_y for I_x.

$$ET_c = \left(I_{y\,et1} - I_{z\,et1}\frac{I_{y\,et0}}{I_{z\,et0}}\right) + \left(I_{y\,et1} - I_{z\,et1}\frac{I_{y\,et0}}{I_{z\,et0}}\right) \tag{11}$$

$$\mathrm{ET_c} = 2I_{\mathrm{y\ et1}} - 2I_{\mathrm{z\ et1}}\frac{I_{\mathrm{y\ et0}}}{I_{\mathrm{z\ et0}}} \tag{12}$$

The axis terms are usually substituted by notations indicating polarization relative to the excitation light. For instance, Supplementary Equation 7 for polarization (P) would have substitutions $I_{\perp} = I_{\mathrm{y}}$ and $I_{||} = I_{\mathrm{z}}$.

$$P = \frac{I_{||} - I_{\perp}}{I_{||} + I_{\perp}} \tag{13}$$

Using the same substitutions and accounting for the total isotropic fluorescence leads to the familiar equation for anisotropy.

$$r = \frac{I_{||} - I_{\perp}}{I_{||} + 2I_{\perp}} \tag{14}$$

Similar substitutions can be made in Supplementary Equation 12 for $\mathrm{ET_c}$.

$$\mathrm{ET_c} = 2I_{\perp\mathrm{et1}} - 2I_{||\mathrm{et1}}\frac{I_{\perp\mathrm{et0}}}{I_{||\mathrm{et0}}} \tag{15}$$

To complete our analogy with a sensitized emission experiment, we need to combine the anisotropy terms with a FRET efficiency equation from [1], where $\mathrm{ET_c}$ represents the corrected energy transfer signal, I_{d} represents the donor fluorescence in the donor channel, and G is a factor relating the sensitized emission signal to the loss in donor fluorescence. G must be determined for each donor-acceptor pairing on each microscope to provide an accurate FRET measurement. This factor accounts for light transmission differences in the emission pathways as well as differences in quantum efficiencies for the donor and acceptor.

$$E = \frac{F_{\mathrm{c}}/G}{I_{\mathrm{d}} + F_{\mathrm{c}}/G} \tag{16}$$

Continuing with our sensitized emission analogy, we must also relate the signal observed in the FRET channel ($I_{\perp}$) to the signal in the donor channel ($I_{||}$). The $I_{||}$ and $I_{\perp}$ emission pathways for an instrument may be equivalent with the exception of the polarizer orientation, but optical components can introduce a polarization bias which makes anisotropy measurements inaccurate. To compensate, a factor, called g, describes any polarization bias in the instrument and is generally determined for any anisotropy experiment. It is most easily determined using small, freely diffusing and rotating fluorescent molecules which should display no polarization bias and the determined g is used to scale the intensity $I_{\perp}$ to that of $I_{||}$. Thus, the fully corrected anisotropy equations also have the g factor, which we insert into Supplementary Equations 14 and 15.

$$r = \frac{I_{||} - gI_{\perp}}{I_{||} + 2gI_{\perp}} \tag{17}$$

$$\mathrm{ET_c} = 2gI_{\perp\mathrm{et1}} - 2I_{||\mathrm{et1}}\frac{gI_{\perp\mathrm{et0}}}{I_{||\mathrm{et0}}} \tag{18}$$

Since the g factor determined for anisotropy measurements adequately compensates for instrument related signal differences in $I_{\perp}$ and $I_{||}$ and the donor and acceptor are the same type of molecule with no differences in extinction coefficient or quantum yield, the G factor normally associated with FRET experiments in Supplementary Equation 14 is unnecessary. Thus, we have

$$E = \frac{\mathrm{ET_c}}{I_{\mathrm{d}} + \mathrm{ET_c}} \tag{19}$$

into which we can substitute our donor channel signal ($I_{||\mathrm{et1}}$) and our corrected energy transfer signal ($\mathrm{ET_c}$).

$$E = \frac{2gI_{\perp\mathrm{et1}} - 2I_{||\mathrm{et1}}\frac{gI_{\perp\mathrm{et0}}}{I_{||\mathrm{et0}}}}{I_{||\mathrm{et1}} + 2gI_{\perp\mathrm{et1}} - 2I_{||\mathrm{et1}}\frac{gI_{\perp\mathrm{et0}}}{I_{||\mathrm{et0}}}} \tag{20}$$

Here, we must point out where our analogy with a hetero-FRET sensitized emission experiment markedly diverges and we introduce a term to replace E for the energy transfer efficiency we are reporting. Treating anisotropy measurements in this manner cannot detect all homo-FRET energy transfer between molecules since the molecules with parallel dipole orientations will undergo efficient energy transfer but will not produce a detectable difference anisotropy. Moreover, any homo-FRET signal in the parallel channel is treated as non-FRET signal. Thus, the energy transfer indicated in Supplementary Equation 20 is actually a conversion of the perpendicular channel signal into a value representing the percent signal change. Although we consider delta r FRET (drFRET) to be akin to hetero-FRET efficiencies, it is denoted differently here since we report only on the increased perpendicular channel signal and we wish to avoid confusion when comparing FRET efficiencies for the same protein-protein interactions using homo-FRET versus hetero-FRET approaches. Therefore, substitution into Supplementary Equation 20 provides the following.

$$\mathrm{drFRET} = \frac{2gI_{\perp\mathrm{et1}} - 2I_{||\mathrm{et1}}\frac{gI_{\perp\mathrm{et0}}}{I_{||\mathrm{et0}}}}{I_{||\mathrm{et1}} + 2gI_{\perp\mathrm{et1}} - 2I_{||\mathrm{et1}}\frac{gI_{\perp\mathrm{et0}}}{I_{||\mathrm{et0}}}} \tag{21}$$

While this equation could perhaps be used directly, relating measured anisotropies to energy transfer simplify analyses. Consider Supplementary Equation 17. Simple rearrangement shows how the ratio of $I_{\perp}$ and $I_{||}$ relate to a measured *r*.

$$rI_{||} + r2gI_{\perp} = I_{||} - gI_{\perp} \tag{22}$$

$$gI_{\perp} + r2gI_{\perp} = I_{||} - rI_{||} \tag{23}$$

$$gI_{\perp}(1 + 2r) = I_{||}(1 - r) \tag{24}$$

$$\frac{gI_{\perp}}{I_{||}} = \frac{(1 - r)}{(1 + 2r)} \tag{25}$$

$$gI_{\perp} = I_{||}\frac{(1 - r)}{(1 + 2r)} \tag{26}$$

The relationships described in Supplementary Equations 25 and 26 should hold for both the non-FRETing control ($_{\mathrm{et0}}$) and the experimental ($_{\mathrm{et1}}$). Designating the variables accordingly produces two equations

$$\frac{gI_{\perp\mathrm{et0}}}{I_{||\mathrm{et0}}} = \frac{(1 - r_{\mathrm{et0}})}{(1 + 2r_{\mathrm{et0}})} \tag{27}$$

$$gI_{\perp\mathrm{et1}} = I_{||\mathrm{et1}}\frac{(1 - r_{\mathrm{et1}})}{(1 + 2r_{\mathrm{et1}})} \tag{28}$$

which can be substituted into Supplementary Equation 21.

$$\mathrm{drFRET} = \frac{2I_{||\mathrm{et1}}\frac{(1 - r_{\mathrm{et1}})}{(1 + 2r_{\mathrm{et1}})} - 2I_{||\mathrm{et1}}\frac{(1 - r_{\mathrm{et0}})}{(1 + 2r_{\mathrm{et0}})}}{I_{||\mathrm{et1}} + 2I_{||\mathrm{et1}}\frac{(1 - r_{\mathrm{et1}})}{(1 + 2r_{\mathrm{et1}})} - 2I_{||\mathrm{et1}}\frac{(1 - r_{\mathrm{et0}})}{(1 + 2r_{\mathrm{et0}})}} \tag{29}$$

Factor out $I_{||\mathrm{et1}}$.

$$\mathrm{drFRET} = \frac{I_{||\mathrm{et1}}(2\frac{(1 - r_{\mathrm{et1}})}{(1 + 2r_{\mathrm{et1}})} - 2\frac{(1 - r_{\mathrm{et0}})}{(1 + 2r_{\mathrm{et0}})})}{I_{||\mathrm{et1}}(1 + 2\frac{(1 - r_{\mathrm{et1}})}{(1 + 2r_{\mathrm{et1}})} - 2\frac{(1 - r_{\mathrm{et0}})}{(1 + 2r_{\mathrm{et0}})})} \tag{30}$$

The $I_{||\mathrm{et1}}$ in the numerator and denominator cancel. Multiply the terms located in both the numerator and denominator by their coefficients.

$$\mathrm{drFRET} = \frac{\frac{(2-\ 2r_{\mathrm{et1}})}{(1+\ 2r_{\mathrm{et1}})} - \frac{(2-\ 2r_{\mathrm{et0}})}{(1+\ 2r_{\mathrm{et0}})}}{1 + \frac{(2-\ 2r_{\mathrm{et1}})}{(1+\ 2r_{\mathrm{et1}})} - \frac{(2-\ 2r_{\mathrm{et0}})}{(1+\ 2r_{\mathrm{et0}})}} \tag{31}$$

Find a common denominator for the functions in the numerator and denominator by multiplying both by $(1+\ 2r_{\mathrm{et1}})(1+\ 2r_{\mathrm{et0}})/(1+\ 2r_{\mathrm{et1}})(1+\ 2r_{\mathrm{et0}})$.

$$\mathrm{drFRET} = \frac{\frac{(2-\ 2r_{\mathrm{et1}})(1+\ 2r_{\mathrm{et1}})(1+\ 2r_{\mathrm{et0}})}{(1+\ 2r_{\mathrm{et1}})(1+\ 2r_{\mathrm{et1}})(1+\ 2r_{\mathrm{et0}})} - \frac{(2-\ 2r_{\mathrm{et0}})(1+\ 2r_{\mathrm{et1}})(1+\ 2r_{\mathrm{et0}})}{(1+\ 2r_{\mathrm{et0}})(1+\ 2r_{\mathrm{et1}})(1+\ 2r_{\mathrm{et0}})}}{\frac{(1+\ 2r_{\mathrm{et1}})(1+\ 2r_{\mathrm{et0}})}{(1+\ 2r_{\mathrm{et1}})(1+\ 2r_{\mathrm{et0}})} + \frac{(2-\ 2r_{\mathrm{et1}})(1+\ 2r_{\mathrm{et1}})(1+\ 2r_{\mathrm{et0}})}{(1+\ 2r_{\mathrm{et1}})(1+\ 2r_{\mathrm{et1}})(1+\ 2r_{\mathrm{et0}})} - \frac{(2-\ 2r_{\mathrm{et0}})(1+\ 2r_{\mathrm{et1}})(1+\ 2r_{\mathrm{et0}})}{(1+\ 2r_{\mathrm{et0}})(1+\ 2r_{\mathrm{et1}})(1+\ 2r_{\mathrm{et0}})}} \tag{32}$$

Several terms will cancel to give a common denominator.

$$\mathrm{drFRET} = \frac{\frac{(2-\ 2r_{\mathrm{et1}})\cancel{(1+\ 2r_{\mathrm{et1}})}(1+\ 2r_{\mathrm{et0}})}{\cancel{(1+\ 2r_{\mathrm{et1}})}(1+\ 2r_{\mathrm{et1}})(1+\ 2r_{\mathrm{et0}})} - \frac{(2-\ 2r_{\mathrm{et0}})(1+\ 2r_{\mathrm{et1}})\cancel{(1+\ 2r_{\mathrm{et0}})}}{(1+\ 2r_{\mathrm{et0}})(1+\ 2r_{\mathrm{et1}})\cancel{(1+\ 2r_{\mathrm{et0}})}}}{\frac{(1+\ 2r_{\mathrm{et1}})(1+\ 2r_{\mathrm{et0}})}{(1+\ 2r_{\mathrm{et1}})(1+\ 2r_{\mathrm{et0}})} + \frac{(2-\ 2r_{\mathrm{et1}})\cancel{(1+\ 2r_{\mathrm{et1}})}(1+\ 2r_{\mathrm{et0}})}{(1+\ 2r_{\mathrm{et1}})\cancel{(1+\ 2r_{\mathrm{et1}})}(1+\ 2r_{\mathrm{et0}})} - \frac{(2-\ 2r_{\mathrm{et0}})\cancel{(1+\ 2r_{\mathrm{et1}})}(1+\ 2r_{\mathrm{et0}})}{(1+\ 2r_{\mathrm{et0}})\cancel{(1+\ 2r_{\mathrm{et1}})}(1+\ 2r_{\mathrm{et0}})}} \tag{33}$$

$$\mathrm{drFRET} = \frac{\frac{(2-\ 2r_{\mathrm{et1}})(1+\ 2r_{\mathrm{et0}}) -\ (2-\ 2r_{\mathrm{et0}})(1+\ 2r_{\mathrm{et1}})}{(1+\ 2r_{\mathrm{et1}})(1+\ 2r_{\mathrm{et0}})}}{\frac{(1+\ 2r_{\mathrm{et1}})(1+\ 2r_{\mathrm{et0}}) + (2-\ 2r_{\mathrm{et1}})(1+\ 2r_{\mathrm{et0}}) - (2-\ 2r_{\mathrm{et0}})(1+\ 2r_{\mathrm{et1}})}{(1+\ 2r_{\mathrm{et1}})(1+\ 2r_{\mathrm{et0}})}} \tag{34}$$

The division of these fractions will cancel more terms.

$$\mathrm{drFRET} = \frac{\frac{(2-\ 2r_{\mathrm{et1}})(1+\ 2r_{\mathrm{et0}}) -\ (2-\ 2r_{\mathrm{et0}})(1+\ 2r_{\mathrm{et1}})}{\cancel{(1+\ 2r_{\mathrm{et1}})(1+\ 2r_{\mathrm{et0}})}}}{\frac{(1+\ 2r_{\mathrm{et1}})(1+\ 2r_{\mathrm{et0}}) + (2-\ 2r_{\mathrm{et1}})(1+\ 2r_{\mathrm{et0}}) - (2-\ 2r_{\mathrm{et0}})(1+\ 2r_{\mathrm{et1}})}{\cancel{(1+\ 2r_{\mathrm{et1}})(1+\ 2r_{\mathrm{et0}})}}} \tag{35}$$

$$\mathrm{drFRET} = \frac{(2-\ 2r_{\mathrm{et1}})(1+\ 2r_{\mathrm{et0}}) -\ (2-\ 2r_{\mathrm{et0}})(1+\ 2r_{\mathrm{et1}})}{(1+\ 2r_{\mathrm{et1}})(1+\ 2r_{\mathrm{et0}}) + (2-\ 2r_{\mathrm{et1}})(1+\ 2r_{\mathrm{et0}}) - (2-\ 2r_{\mathrm{et0}})(1+\ 2r_{\mathrm{et1}})} \tag{36}$$

Use the FOIL method to multiply the binomial expressions in the numerator and denominator.

$$\mathrm{drFRET} = \frac{2 + 4r_{\mathrm{et0}} - 2r_{\mathrm{et1}} - 4r_{\mathrm{et0}}r_{\mathrm{et1}} -\ (2 + 4r_{\mathrm{et1}} -\ 2r_{\mathrm{et0}} - 4r_{\mathrm{et0}}r_{\mathrm{et1}})}{1 + 2r_{\mathrm{et0}} + 2r_{\mathrm{et1}} + 4r_{\mathrm{et0}}r_{\mathrm{et1}} + 2 + 4r_{\mathrm{et0}} - 2r_{\mathrm{et1}} - 4r_{\mathrm{et0}}r_{\mathrm{et1}} -\ (2 + 4r_{\mathrm{et1}} -\ 2r_{\mathrm{et0}} - 4r_{\mathrm{et0}}r_{\mathrm{et1}})} \tag{37}$$

Rearrange while accounting for the negative coefficient for the last binomial multiplication in both the numerator and denominator.

$$\mathrm{drFRET} = \frac{\cancel{2-2} + 4r_{\mathrm{et0}} +\ 2r_{\mathrm{et0}} - 2r_{\mathrm{et1}} - 4r_{\mathrm{et1}} - \cancel{4r_{\mathrm{et0}}r_{\mathrm{et1}} + 4r_{\mathrm{et0}}r_{\mathrm{et1}}}}{1 + \cancel{2-2} + 2r_{\mathrm{et0}} + 4r_{\mathrm{et0}} +\ 2r_{\mathrm{et0}} + \cancel{2r_{\mathrm{et1}} - 2r_{\mathrm{et1}}} - 4r_{\mathrm{et1}} + 4r_{\mathrm{et0}}r_{\mathrm{et1}} \cancel{- 4r_{\mathrm{et0}}r_{\mathrm{et1}} + 4r_{\mathrm{et0}}r_{\mathrm{et1}}}} \tag{38}$$

Cancel and combine several of the terms.

$$\mathrm{drFRET} = \frac{6r_{\mathrm{et0}} - 6r_{\mathrm{et1}}}{1 + 8r_{\mathrm{et0}} - 4r_{\mathrm{et1}} + 4r_{\mathrm{et0}}r_{\mathrm{et1}}} \tag{39}$$

$$\mathrm{drFRET} = \frac{6(r_{\mathrm{et0}} - r_{\mathrm{et1}})}{1 + 8r_{\mathrm{et0}} - 4r_{\mathrm{et1}} + 4r_{\mathrm{et0}}r_{\mathrm{et1}}} \tag{40}$$

The final Supplementary Equation 40 comes with at least one caveat. The anisotropy for the control should represent the anisotropy of the molecule of interest in the absence of FRET. While this seems obvious, it may not be practical to determine this value for some molecules. For instance, a fluorescently labeled protein may have different anisotropies as a monomer versus a dimer even in the absence of homoFRET since the rotational dynamics of the dimer might be different compared to the monomer. In such an example, the dimer in the absence of FRET would be expected to have a higher anisotropy. The presence of homo-FRET would reduce the anisotropy to r_{et1}, but using the r_{et0} value determined from a monomer would underestimate the energy transfer. Such a small difference may have little consequence if one wishes only to compare FRET efficiencies measured across laboratories under numerous conditions, but we feel obligated to point out this potential pitfall.

On the other hand, photobleaching or photoswitching off of the fluorescent tags can provide a readout of the anisotropy of the molecule of interest in the absence of reduced energy transfer. When photoswitched on, anisotropy measurements of photoswitchable fluorescent proteins provide r_{et1} at the initial fluorescence level. As the molecules photoswitch off over time, they will no longer be able to FRET with neighboring molecules. Thus, the anisotropy will increase to conditions closely resembling a true $\mathrm{r_{et0}}$, where the measured anisotropy is dictated by rotational motion of the protein complex and is independent of FRET.

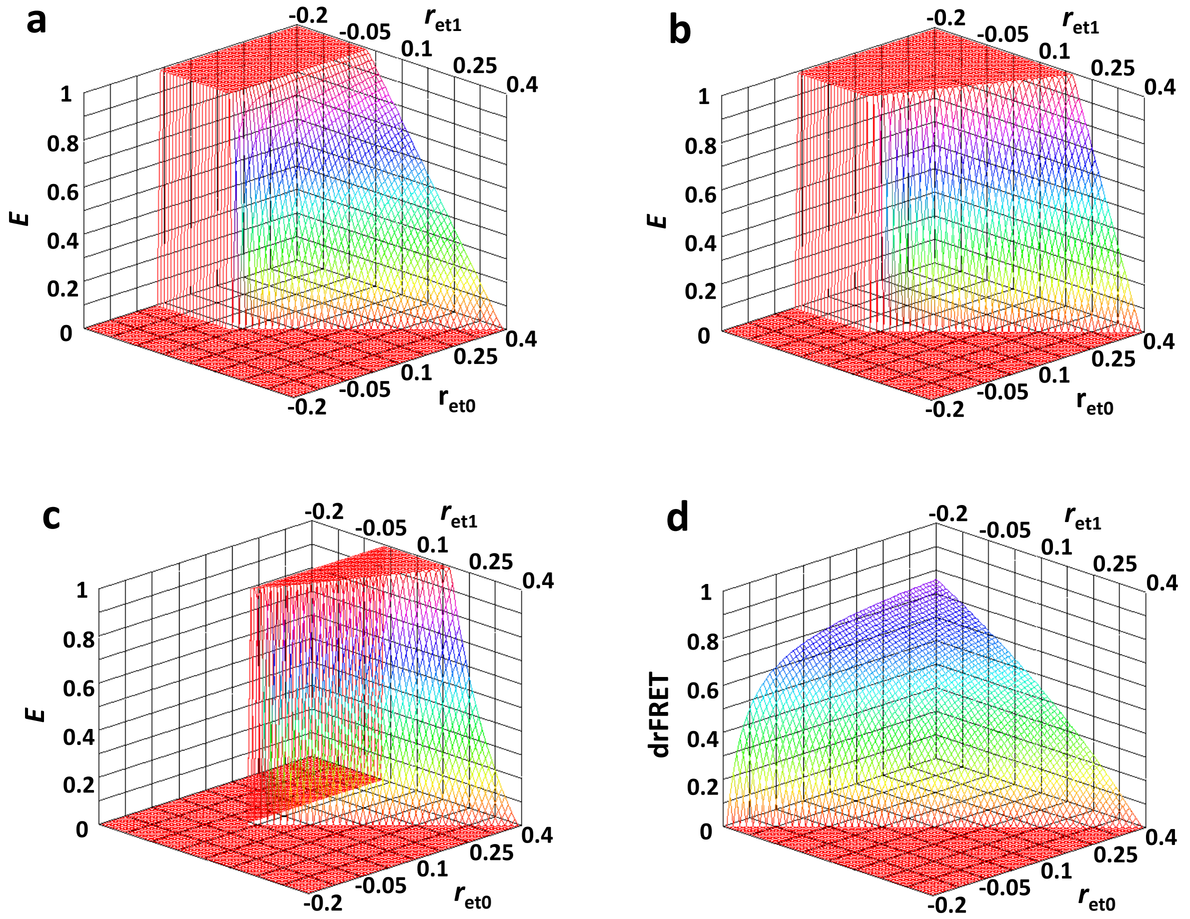

Supplementary Figure 1. Three dimensional graphs show the relationship between FRET efficiency values and changes in anisotropy. Here, we show the range of obtainable values using three equations found within the literature[2-4] and our own Supplementary Equation 40 to convert anisotropy changes to energy transfer efficiency. Here, we assumed one-photon excitation and photoselection of an isotropic oriented population of fluorophores with the minimum and maximum anisotropy values of -0.2 and 0.4, respectively. The equations are as follows (**a**) $E = 1 - \frac{r_c}{r_m}$, where r_c=r_{et1} and r_m=r_{et0}. (**b**) $E = \frac{2(r_{01} - \langle r \rangle)}{r_{01}}$, where $\langle r \rangle$=r_{et1} and r_{01}=r_{et0}. (**c**) $E = \frac{12(r_{max} - r)}{5r(2 + r_{max})}$, where r=r_{et1} and r_{max}=r_{et0}. (**d**) $\text{drFRET} = \frac{6(r_{et0} - r_{et1})}{1 + 8r_{et0} - 4r_{et1} + 4r_{et0}r_{et1}}$.

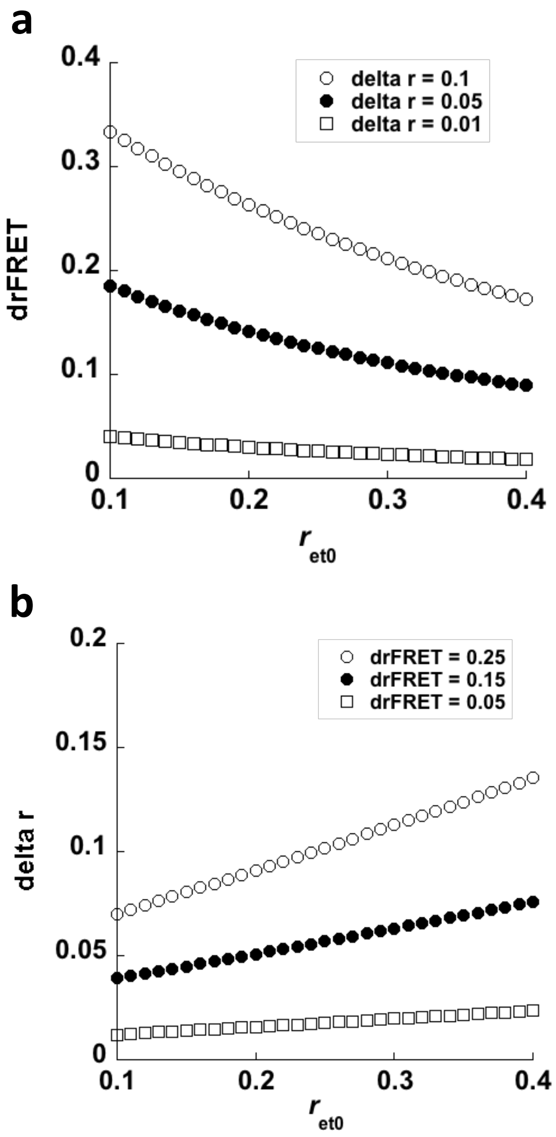

Supplementary Figure 2. The relationships associated with drFRET, r_{et0}, and delta r. (**a**) To readily show the relationship over the range of anisotropy values and changes in anisotropy we observe in our psAFRET experiments, we plot three different delta r values (0.1, 0.05, and 0.01) for a r_{et0} range of 0.1-0.4. This illustrates that a given delta r represents a slightly different drFRET value depending on the r_{et0} value. (**b**) Rearranging Supplementary Equation 40 to isolate r_{et1}, we can calculate the change in anisotropy (delta r) expected for a given r_{et0} and three different drFRET (0.25, 0.15, and 0.05).

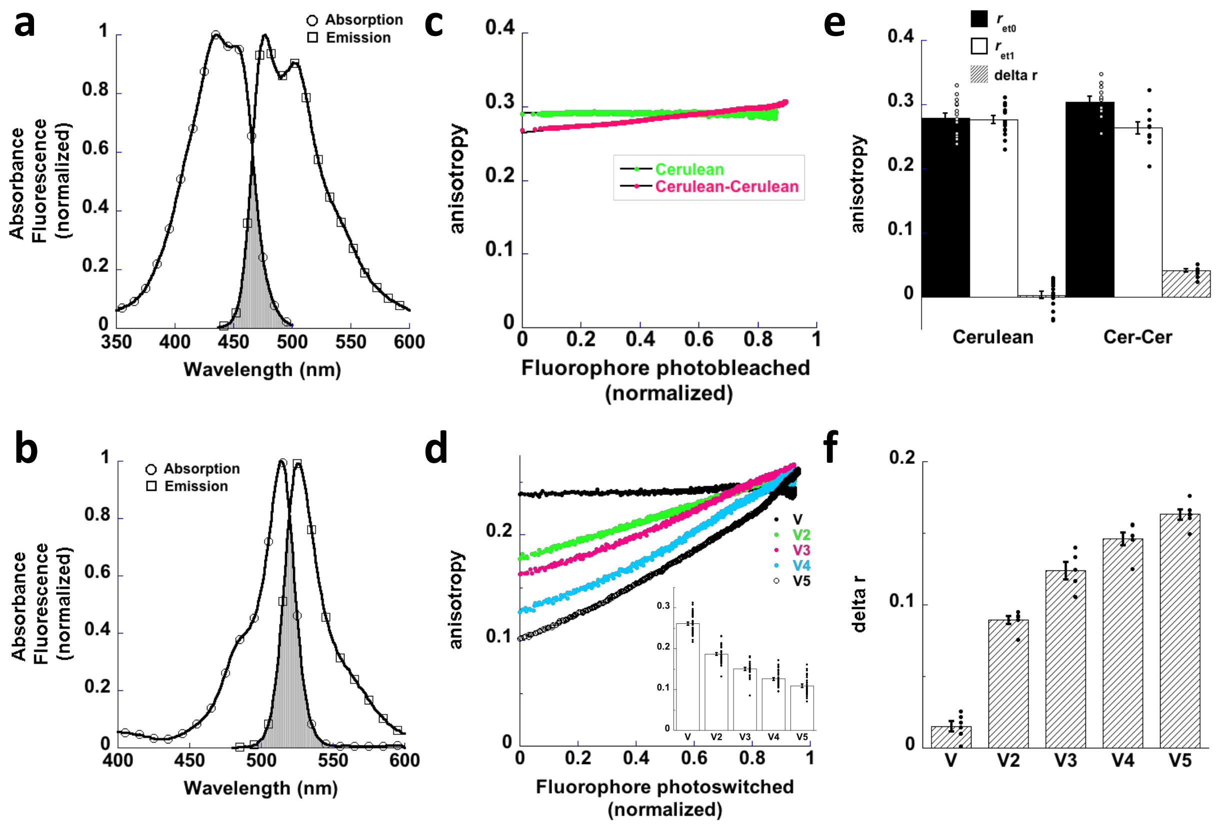

Supplementary Figure 3. Photobleaching anisotropy measurements of Cerulean and Venus fluorescent proteins. (**a**) Absorption (open circles) and emission (open squares) spectra of Cerulean protein are shown. The spectral overlap between the absorption and emission are shown by the gray area. (**b**) Absorption (open circles) and emission (open squares) spectra of Venus are shown. The spectral overlap between the absorption and emission are shown by the gray area. (**c**) COS-7 cells expressing Cerulean (green) or Cerulean-Cerulean (magenta) were imaged and photobleached while collecting parallel and perpendicular polarized fluorescence emission. The anisotropy was determined and displayed as a function of the fluorophore photobleached. The data points representing ~80% of the fluorescence were fitted to a linear equation (black line). (**d**) COS-7 cells expressing Venus (black circles), V2 (green circles), V3 (magenta circles), V4 (blue circles), or V5 (open circles) were imaged and photobleached while collecting parallel and perpendicular polarized fluorescence emission. The anisotropy was determined and displayed as a function of the fluorophore photobleached. The data points were fitted to a linear equation to determine the change in anisotropy. The steady-state anisotropy values for the Venus oligomers were determined from single images of the oligomers expressed in COS-7 cells combined with the data points collected at the onset of the photobleaching experiment (inset; white columns). Data represent mean±sem (n=37, 36, 24, 32, and 29 for V1, V2, V3, V4, and V5, respectively). ANOVA tests indicated significant differences in conventional steady-state anisotropy for all oligomers (p-value <0.05; Cohen's d values ranged from 0.77 – 6.87). (**e**) Linear fits of the data in **c** were used to determine the

anisotropy of Cerulean and Cerulean-Cerulean before photobleaching (white columns), after photobleaching (black columns) and the difference (hatched columns). Data represent mean±sem (n=15 and 11 for Cerulean and Cerulean-Cerulean, respectively). ANOVA tests indicated significant differences between the pre- and post-photobleaching Cerulean-Cerulean anisotropy values (p-value <0.05; Cohen's d = 1.49). Two-tailed t-tests indicated a significant difference in the delta r between Cerulean and Cerulean-Cerulean (p-value <0.05; Cohen's d = 2.27) (**f**) Linear fits were used to determine the change in anisotropy (delta r) of the Venus oligomers during the photobleaching experiment (hatched columns). Data represent mean±sem (n=6, 6, 5, 6, and 6 for V1, V2, V3, V4, and V5, respectively). ANOVA tests indicate significant differences in delta r for all oligomers (p-value <0.05; Cohen's d ranged 1.71 – 14.85). Circles overlaid on columns in the bar graphs represent individual data points. Source data are provided as a Source Data file.

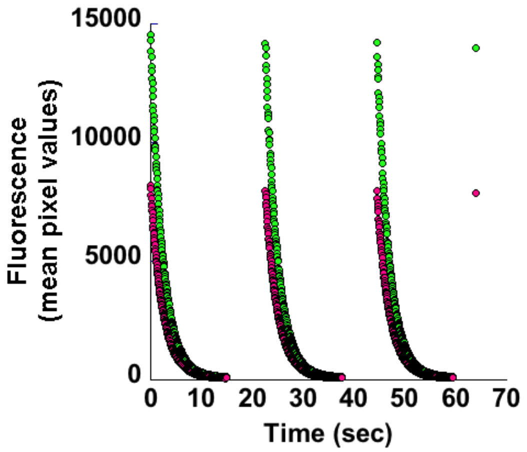

Supplementary Figure 4. Photoswitching anisotropy measurements of Dronpa fluorescent protein. A COS-7 cell expressing Dronpa was excited using 488nm excitation and imaged with parallel and perpendicular emission channels. The fluorescence intensities in the parallel (green) and perpendicular (magenta) are shown. Brief illumination with 405nm light photoswitches Dronpa back to the on state and restores the parallel and perpendicular fluorescence signal. In this example, three photoswitching on-off cycles are shown. Source data are provided as a Source Data file.

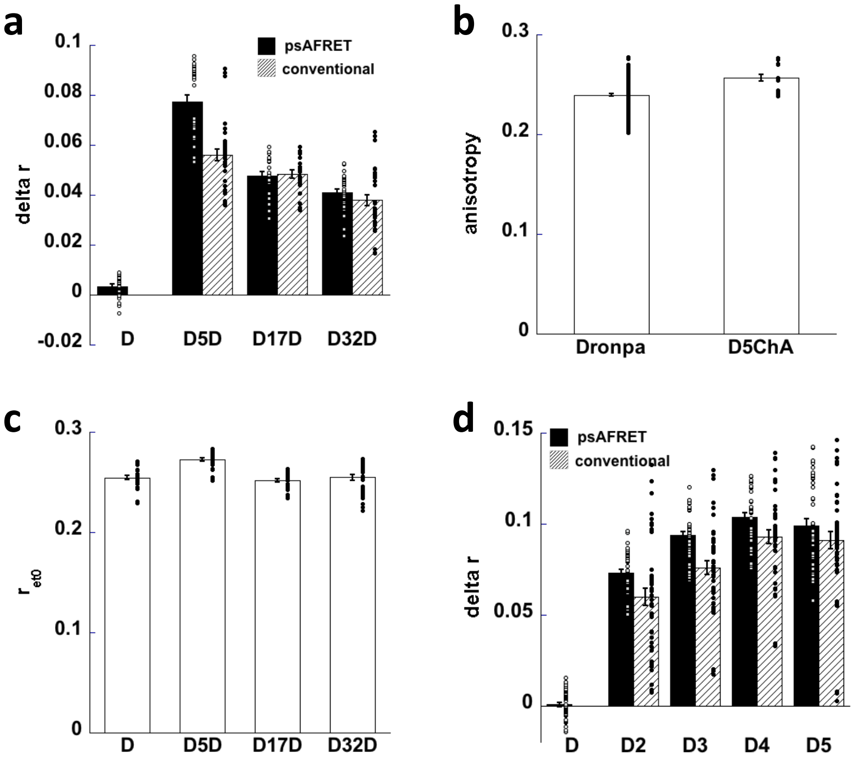

Supplementary Figure 5. Comparisons of delta r calculations made from psAFRET experiments and conventional anisotropy calculations. Conventional anisotropy measurements can be made on psAFRET data using the parallel and perpendicular channel intensities from the first image of a photoswitching experiment. (**a**) The delta r values determined from psAFRET for Dronpa, D5D, D17D, and D32D in figure 2 of the main text (black columns) are compared here with the delta r values determined from conventional calculations using Dronpa alone as the non-FRET control (hatched columns). Data represent mean ± sem (n= 30, 33, 23, and 33 for D, D5D, D17D, and D32D, respectively). ANOVA tests indicated significant differences between psAFRET and conventional delta r values for D5D (p-value <0.05; Cohen's d = 1.47). (**b**) Conventional steady-state anisotropy determinations for Dronpa alone are compared with the steady-state anisotropy for Dronpa-5-mCherryAmber (D5ChA), a non-FRET control chimera which should be approximately the same size as D5D. Data represent mean ± sem (n=159 and 18, respectively). Two-tailed t-tests indicated a significant difference between Dronpa and D5ChA (p-value <0.05; Cohen's d = 4.67). (**c**) The psAFRET analyses produces estimates of r_{et0}, which is the anisotropy in the absence of energy transfer. These are plotted for the Dronpa, D5D, D17D, and D32D chimeras discussed in figure 2 of the main text. Data represent mean ± sem (n= 30, 33, 23, and 33 for D, D5D, D17D, and D32D, respectively). ANOVA tests indicated significant differences between D5D and the other chimeras (p-value <0.05; Cohen's d ranged from 1.49 – 1.76). (**d**) The delta r values determined from psAFRET for Dronpa, D2, D3, D4, and D5 in figure 3 of the main text (black columns) are compared here with the delta r values determined from conventional calculations using Dronpa alone as the non-FRET control (hatched columns). ANOVA tests indicated significant differences between psAFRET and conventional delta r values for D2, D3, and D4 (p-value <0.05; Cohen's d ranged 0.51 – 0.79). Data represent mean ± sem (n=45 for each). Circles overlaid on columns in the bar graphs represent individual data points. Source data are provided as a Source Data file.

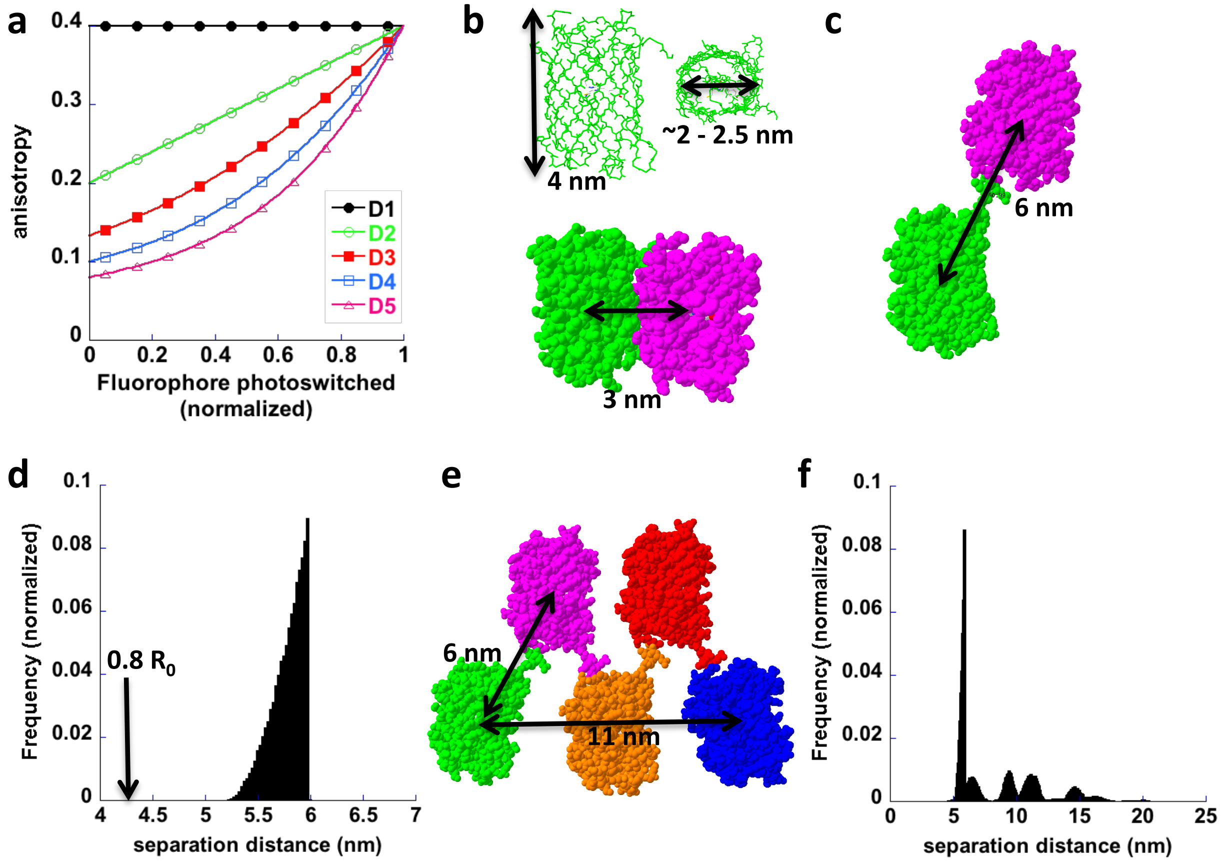

Supplementary Figure 6. Simulations of photoswitching anisotropy measurements of Dronpa oligomers. The anisotropy of Dronpa oligomers, Dronpa (filled black circles), D2 (open green circles), D3 (filled red squares), D4 (open blue squares), and D5 (open magenta triangles) were simulated as a function of fluorophore photoswitched. Based on the studies of Runnels and Scarlata[5], the expected changes in anisotropy as a function of cluster size are heavily dependent on the ratio of the separation distance to R_0. Thus, it is noteworthy that the upward curvatures of the photoswitching or photobleaching profiles depend on the separation distance between the molecules being ≤ 0.8 R_0 [6]. (**a**) The oligomers were simulated using a binomial distribution described in Yeow and Clayton[6]. Dronpa has an R_0 ~5.3nm and an assumption for this approach is that the average distance between the fluorophores is 0.8*R_0. (**b**) The backbone beta-barrel structure of Dronpa molecule is shown to be approximately 4 nm in length and 2 – 2.5 nm in diameter[7]. However, inclusion of the side chains and space filling models suggest that likely the closest distance between two fluorophores will be ~3nm. (**c**) For this example, two Dronpa molecules are linked with a 5-amino acid linker and moved as far apart as possible. The chromophore to chromophore distance could reach ~6 nm. As noted previously[8,9], assuming an average κ^2=2/3 for fluorescent proteins is not valid but would represent the most liberal estimate and result in the highest possible R_0. Assuming this best case scenario and using the overlap integrals between the absorption and emission spectra for Dronpa and Venus, we calculated the Dronpa-Dronpa and Venus-Venus R_0 values to be ~5.3nm. Therefore, the chromophores for our oligomer chimeras would need to be within ~4.25 nm

to meet ≤0.8 R_0 criteria. (**d**) Simulations were performed in which 100,000 Dronpa molecules are randomly assembled into dimers with a limitation of 3nm minimum distance and 6nm maximum distance between the chromophores. The arrow indicates the approximate 0.8*R_0 for Dronpa homo-FRET. (**e**) For this example, five Dronpa molecules are linked with 5-amino acid linkers and are shown in one potential configuration. This suggests that the distances between each of the chromophores can be variable for a Dronpa5 chimera. (**f**) Simulations were performed in which 100,000 Dronpa molecules are randomly assembled into pentamers with a limitation of 3nm minimum distance and 6nm maximum distance between each chromophore in the linear sequence. These show an even broader distribution with the separation distances up to ~20 nm for each molecule in the chain. Dronpa images were produced using the Swiss PDB Viewer using the Dronpa structure pdb file 2IE2 [7].

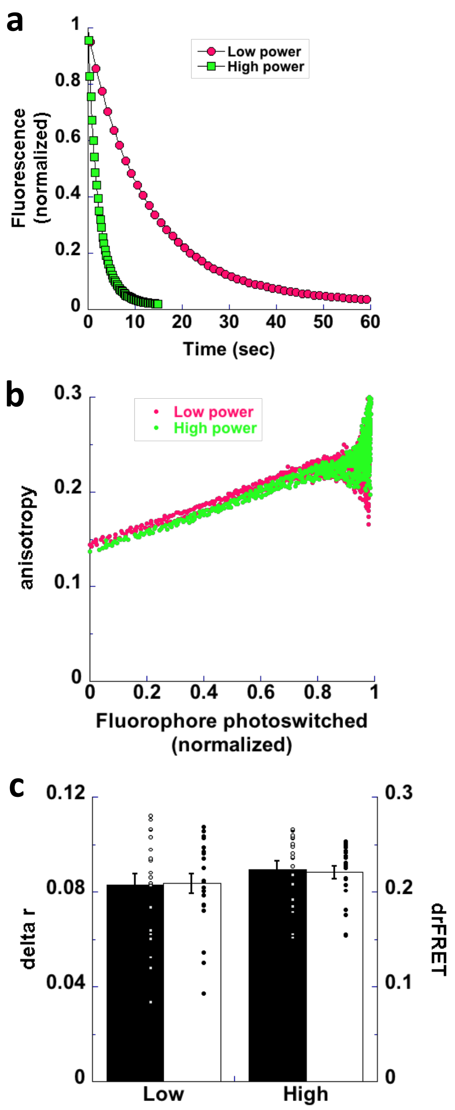

Supplementary Figure 7. Illumination dependence of Dronpa oligomer psAFRET measurements. (**a**) COS-7 cells expressing D5 were excited using 488nm excitation under high (green squares) or low (magenta circles) laser intensity and imaged with parallel and perpendicular emission channels. The total fluorescence intensities were normalized to the initial fluorescence and are displayed as Dronpa is photoswitched off. (**b**) The anisotropy was determined and displayed as a function of the fluorophore photoswitched under high (green circles) and low (magenta circles) 488nm laser illumination. (**c**) The data points representing ~80% of the fluorescence were fitted to linear equations. These were used to determine the anisotropy before and after photoswitching and the difference in anisotropy (delta r, black columns) is shown compared to its conversion to FRET efficiency (white columns). Data represent mean±sem (n=21 for both low and high power). Two tailed t-tests found no significant differences between the delta r values nor the drFRET values when comparing the low and high laser power experiments. Circles overlaid on columns in the bar graphs represent individual data points. Source data are provided as a Source Data file.

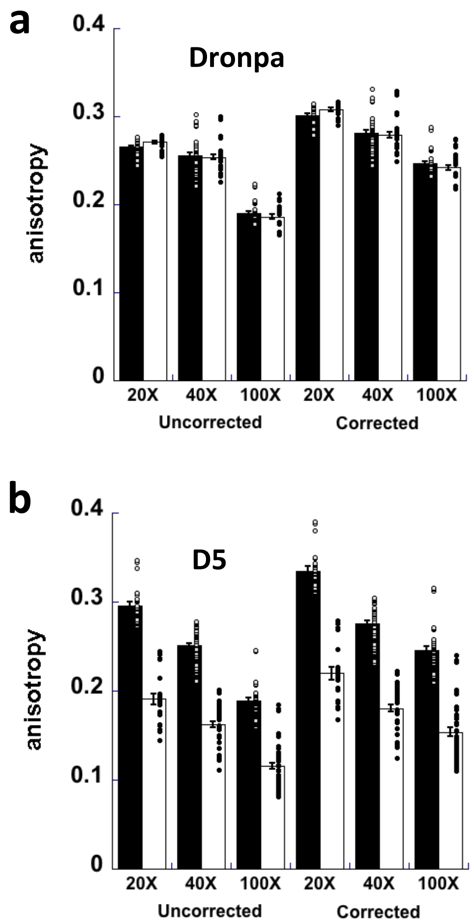

Supplementary Figure 8. Photoswitching anisotropy measurements of Dronpa fluorescent protein oligomers. COS-7 cells expressing (**a**) Dronpa or (**b**) D5 were imaged using either a 20X 0.75 NA, 40X 1.0 NA, or 100X 1.4 NA objective lens as indicated. The anisotropy during photoswitching was determined, plotted as a function of the fluorophore photoswitched, and the data points representing ~80% of the fluorescence were fitted to linear equations. The linear fits were used to determine the anisotropy of Dronpa oligomers before photoswitching (white columns) and after photoswitching (black columns). As indicated, data are uncorrected or corrected for use of high NA objective lenses using the Axelrod correction. For Dronpa measurements (a), ANOVA tests indicated no significant anisotropy differences between pre-photoswitching (white columns) and post-photoswitching (black columns) for each condition. Significant differences were observed between the anisotropy values for the various objectives (p-value <0.05; Cohen's d values ranged from 1.08 – 5.18) except for the uncorrected 20X and 40X pre-photoswitching measurements (white columns). For the D5 measurements (b) significant differences were found in ANOVA comparisons between pre-photoswitching (white columns) and post-photoswitching (black columns) for all conditions as well as significant anisotropy differences in comparisons between objectives (p-value <0.05; Cohen's d values ranged from 0.97 – 7.75). Data represent mean±sem (n=21, 39, and 39 for 20X, 40X, and 100X, respectively in **a**; n=24, 42, and 45 for the 20X, 40X, and 100X, respectively in **b**). Circles overlaid on columns in the bar graphs represent individual data points. Source data are provided as a Source Data file.

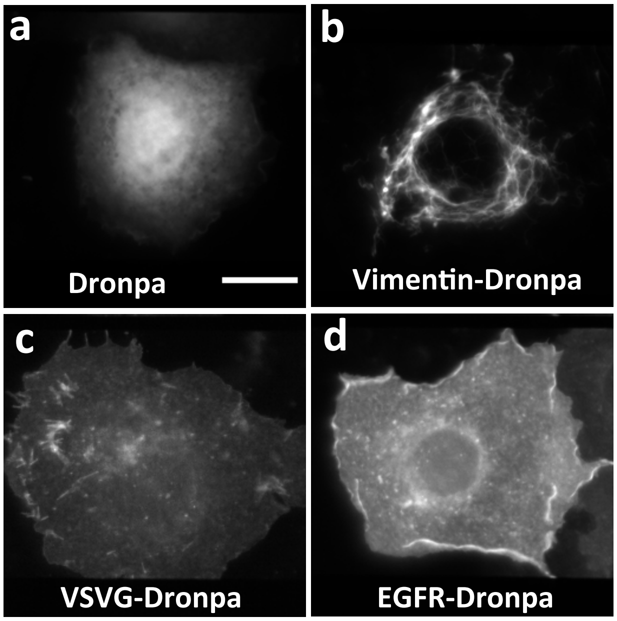

Supplementary Figure 9. Example images of Dronpa tagged proteins. COS-7 cells expressing (**a**) Dronpa, (**b**) Vimentin-Dronpa, (**c**) VSVGtsO45-Dronpa, or (**d**) EGFR-Dronpa were imaged and photoswitched. A subset of EGFR-Dronpa expressing cells were also treated with EGF. These images are derived from the parallel fluorescence emission channel. Scale bar in (**a**) is 10μm and applies to all images.

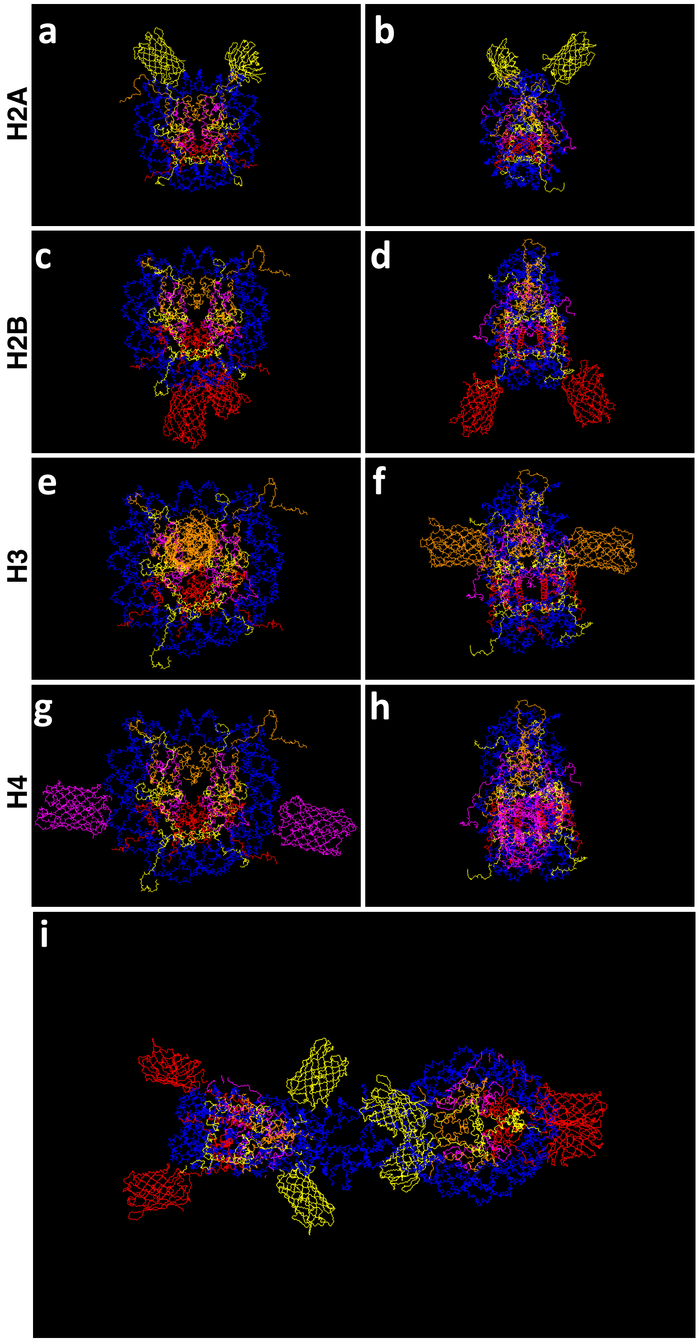

Supplementary Figure 10. Core histone-Dronpa proteins are modeled in a nucleosome. A nucleosome is shown from orthogonal views in which two Dronpa molecules have been depicted as attached to the C termini of (**a**-**b**) H2A, (**c**-**d**) H2B, (**e**-**f**) H3, and (**g**-**h**) H4. These images show the DNA (blue), H2A (yellow), H2B (red), H3 (orange), and H4 (magenta). Images were produced using the Swiss PDB Viewer using the nucleosome structure pdb file 1eqz[10] and the Dronpa structure pdb file 2IE2[7]. (**i**) The tetranucleosome structure pdb file 1zbb[11] was used with pdb file 2IE2 to depict the potential locations of Dronpa attached to the C-termini of H2A (yellow) and H2B (red) during predicted higher order chromatin folding.

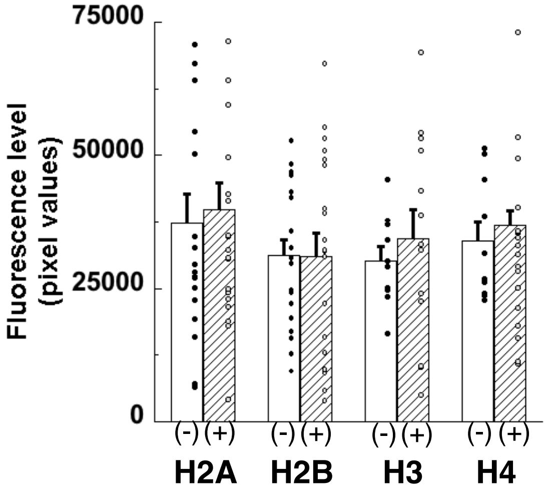

Supplementary Figure 11. Expression levels for Dronpa tagged histones. COS-7 cells expressing H2A-Dronpa, H2B-Dronpa, H3-Dronpa, and H4-Dronpa were imaged and photoswitched. The cells were mock treated (white columns) or treated with Calyculin A (hatched columns) before imaging. The values represent the total fluorescence signal from each cell in pixel values determined from the first image of the photoswitching experiment using $I_{||} + 2gI_{\perp}$. Data represent mean±sem (n=19, 20, 20, 10, 13, 10, and 20 for H2A-, H2A+, H2B-, H2B+, H3-, H3+, H4-, H4+, respectively). Two-tailed t-tests did not indicate a significant difference between the fluorescence levels of the control versus Calyculin A treated cells for any of the histone chimeras. Circles overlaid on columns in the bar graphs represent individual data points. Source data are provided as a Source Data file.

Supplementary Table 1. ANOVA and Tukey-Kramer test (α = 0.05) results for figure 4 in the main text.

Chimera	Objective lens comparison	delta r		drFRET	
		Uncorrected (Cohen's d)	Corrected (Cohen's d)	Uncorrected (Cohen's d)	Corrected (Cohen's d)
4a. D1	20X-40X	1.17	1.15	1.08	1.05
	20X-100X	1.36	1.41	1.39	1.4
	40X-100X	n.s.	n.s.	n.s.	n.s.
4b. D5D	20X-40X	n.s.	n.s.	n.s.	n.s.
	20X-100X	1.37	0.96	n.s.	n.s.
	40X-100X	1.09	n.s.	n.s.	n.s.
4c. D32D	20X-40X	0.88	0.91	0.69	0.66
	20X-100X	1.22	0.81	n.s.	n.s.
	40X-100X	n.s.	n.s.	n.s.	n.s.
4d. D5	20X-40X	0.89	0.92	n.s.	n.s.
	20X-100X	1.74	1.09	n.s.	n.s.
	40X-100X	0.85	n.s.	n.s.	n.s.

n.s. = no significant difference observed

Supplementary Table 2. List of primers used in this study.

Primer name	Sequence (5’---3’)
Cerulean_5_Bgl2	GATCAGATCTGTGAGCAAGGGCGAGGAG
Cerulean_3_EcoR1	GATCGAATTCCTTGTACAGCTCGTCCAT
Dronpa_5_Bgl2	GATCAGATCTAGTGTGATTAAACCAGAC
Dronpa_3_EcoR1	GATCGAATTCCTTGGCCTGCCTCGGCAG
Dronpa_5_Kpn1	GATCGGTACCATGAGTGTGATTAAACCAG
Dronpa_3_BamH1	GATCGGATCCTTACTTGGCCTGCCTCGG
D3_5_Sal1	GATCGTCGACGGGTGAGCAAGGGCGAGGAG
D3_3_BamH1	GATCGGATCCCTTGTACAGCTCGTCCAT
D4_5_BamH1	GATCGGATCCACTGGAACTAGTGTGATTAAACCAG
D4_3_Xba1	GATCTCTAGACTTGGCCTGCCTCGGC
D5_5_BspE1	GATCTCCGGAACTGGAACTAGTGTGATTAAACCAG
D5_3_Bgl2	GATCAGATCTAGTTCCAGTCTTGGCCTGCCTCGGC

Supplementary References

1 Chen, H., Puhl, H. L., 3rd, Koushik, S. V., Vogel, S. S. & Ikeda, S. R. Measurement of FRET efficiency and ratio of donor to acceptor concentration in living cells. *Biophys J* **91**, L39-41, doi:10.1529/biophysj.106.088773 (2006).

2 Ghosh, S., Saha, S., Goswami, D., Bilgrami, S. & Mayor, S. Dynamic imaging of homo-FRET in live cells by fluorescence anisotropy microscopy. *Methods Enzymol* **505**, 291-327, doi:10.1016/B978-0-12-388448-0.00024-3 (2012).

3 Hamman, B. D., Oleinikov, A. V., Jokhadze, G. G., Traut, R. R. & Jameson, D. M. Dimer/monomer equilibrium and domain separations of Escherichia coli ribosomal protein L7/L12. *Biochemistry* **35**, 16680-16686, doi:10.1021/bi9624189 (1996).

4 Snell, N. E. *et al.* Homotransfer FRET Reporters for Live Cell Imaging. *Biosensors-Basel* **8**, doi:ARTN 89 10.3390/bios8040089 (2018).

5 Runnels, L. W. & Scarlata, S. F. Theory and application of fluorescence homotransfer to melittin oligomerization. *Biophys J* **69**, 1569-1583, doi:10.1016/S0006-3495(95)80030-5 (1995).

6 Yeow, E. K. & Clayton, A. H. Enumeration of oligomerization states of membrane proteins in living cells by homo-FRET spectroscopy and microscopy: theory and application. *Biophys J* **92**, 3098-3104, doi:10.1529/biophysj.106.099424 (2007).

7 Wilmann, P. G. *et al.* The 1.7 A crystal structure of Dronpa: a photoswitchable green fluorescent protein. *J Mol Biol* **364**, 213-224, doi:10.1016/j.jmb.2006.08.089 (2006).

8 Vogel, S. S., Nguyen, T. A., van der Meer, B. W. & Blank, P. S. The impact of heterogeneity and dark acceptor states on FRET: implications for using fluorescent protein donors and acceptors. *PLoS One* **7**, e49593, doi:10.1371/journal.pone.0049593 (2012).

9 van der Meer, B. W., van der Meer, D. M. & Vogel, S. S. in *FRET - Förster Resonance Energy Transfer From Theory to Applications* (eds I. Medintz & N. Hildebrandt) (Wiley-VCH, 2014).

10 Harp, J. M., Hanson, B. L., Timm, D. E. & Bunick, G. J. Asymmetries in the nucleosome core particle at 2.5 A resolution. *Acta Crystallogr D Biol Crystallogr* **56**, 1513-1534 (2000).

11 Schalch, T., Duda, S., Sargent, D. F. & Richmond, T. J. X-ray structure of a tetranucleosome and its implications for the chromatin fibre. *Nature* **436**, 138-141, doi:10.1038/nature03686 (2005).
